# Shotgun-based proteomics of extracellular vesicles in Alzheimer’s disease reveals biomarkers involved in immunological and coagulation pathways

**DOI:** 10.1038/s41598-021-97969-y

**Published:** 2021-09-16

**Authors:** Jonas Ellegaard Nielsen, Bent Honoré, Karsten Vestergård, Raluca Georgiana Maltesen, Gunna Christiansen, Anna Uhd Bøge, Søren Risom Kristensen, Shona Pedersen

**Affiliations:** 1 Department of Clinical Medicine, Aalborg University, 9000 Aalborg, Denmark; 2 Department of Clinical Biochemistry, Aalborg University Hospital, 9000 Aalborg, Denmark; 3 Department of Biomedicine, Aarhus University, 8000 Aarhus, Denmark; 4 Department of Neurology, Aalborg University Hospital, 9000 Aalborg, Denmark; 5 Translational Radiation Biology and Oncology Laboratory, Centre for Cancer Research, Westmead Institute of Medical Research, 2145 Sydney, Australia; 6 Department of Anaesthesia and Intensive Care, Aalborg University Hospital, 9000 Aalborg, Denmark; 7 Department of Health Science and Technology, Aalborg University, 9220 Aalborg, Denmark; 8 Department of Basic Medical Sciences, College of Medicine, Qatar University, 2713 Doha, Qatar

**Keywords:** Proteomics, Diagnostic markers, Alzheimer's disease

## Abstract

Alzheimer’s disease (AD) is the most common form of dementia and without readily available clinical biomarkers. Blood-derived proteins are routinely used for diagnostics; however, comprehensive plasma profiling is challenging due to the dynamic range in protein concentrations. Extracellular vesicles (EVs) can cross the blood–brain barrier and may provide a source for AD biomarkers. We investigated plasma-derived EV proteins for AD biomarkers from 10 AD patients, 10 Mild Cognitive Impairment (MCI) patients, and 9 healthy controls (Con) using liquid chromatography-tandem mass spectrometry (LC–MS/MS). The ultracentrifuged EVs were washed and confirmed according to the MISEV2018 guidelines. Some AD patients presented with highly elevated *FXIIIA1* (log_2_ FC: 4.6, *p*-value: 0.005) and *FXIIIB* (log_2_ FC: 4.9, *p*-value: 0.018). A panel of proteins was identified discriminating Con from AD (AUC: 0.91, CI: 0.67–1.00) with *ORM2* (AUC: 1.00, CI: 1.00–1.00), *RBP4* (AUC: 0.99, CI: 0.95–1.00), and *HYDIN* (AUC: 0.89, CI: 0.72–1.00) were found especially relevant for AD. This indicates that EVs provide an easily accessible matrix for possible AD biomarkers. Some of the MCI patients, with similar protein profiles as the AD group, progressed to AD within a 2-year timespan.

## Introduction

Alzheimer’s disease (AD) is the main contributor to the group of dementia types, with a multitude of underlying mechanisms, urgently requiring improved understanding and comprehension^1^. The more investigated pathways include amyloid-β (Aβ) deposition, vascular complications, and inflammation, with these pathways assumed to be implicated in risk of AD, and contributing to disease pathology and progression^1,2^. With an increasingly aging population, there is mounting pressure to identify these pathophysiological mechanisms and their related biomarkers^3^.

Current diagnostic methods, including advanced imaging techniques and measurements of cerebrospinal fluid (CSF) proteins, are impeded by limitations such as affordability, invasiveness, and accessibility in clinical practices^4^. It would therefore be advantageous to identify non-invasive biomarkers indicative of the disease stage. The use of blood-based biomarkers may increase patient compliance and the cost-effectiveness of AD diagnostics. Furthermore, the blood–brain barrier (BBB) is believed to be compromised at earlier stages of AD pathogenesis, thereby allowing larger molecules to leak from the central nervous system (CNS) into the circulation^5^.

These necessities have solicited an unbiased approach, where thousands of molecules are simultaneously explored. Proteomics is often used as an untargeted platform for biomarker discovery^6^; an approach that has already resulted in interesting biomarker candidates for AD, such as neurogranin, neurofilament light chain, TREM2, and YKL-40^3,7,8^. However, failure to replicate results has halted the transition of biomarker candidates from bench to bedside^9^. Blood is a complex source of information, especially for differences in protein concentrations, where the dynamic range can span at least 10 orders of magnitude, from the highly abundant albumin (~ 50 mg/mL) to low abundant cytokines (~ 5 pg/mL)^6^. This dynamic range complicates the extensive profiling using proteomics methods such as mass spectrometry (MS), which is biased towards highly abundant proteins^10^. To partially overcome this obstacle, abundant plasma proteins (up to 22 proteins) are often depleted prior to proteomics analysis. However, several caveats complicate the replicability of these studies, as they introduce variabilities in sample measurements. The caveats include lack of high specificity for antibodies with the removal of non-specific proteins and depletion under denaturing conditions causing co-immunoprecipitation and possible removal of bound proteins^11^. Furthermore, in biomarker studies the presence of confounding factors such as liver and kidney function, and/or other co-morbidities could also affect the reproducibility and interpretation of the results obtained from MS-based proteomics analyses^12^.

In the last decade, extracellular vesicles (EVs) have emerged as important intercellular communicators, regulating both physiological and pathological processes. These vesicles comprise groups of double lipid-layer membranous particles of heterogeneous size and composition. When formed, these entities can be loaded with proteins, lipids, and genetic material such as RNAs and miRNAs. The composition of the EV cargo could therefore reflect the physiological state of the parental cell from where it originated^13^. This important feature has shed light on EVs as novel sources of disease-related biomarkers^14^. EVs can cross the BBB^15^, enabling their release from cells within the CNS, including diseased cells if present and circulate in the blood.

Several studies have investigated biomarkers for AD in EVs; however, their focus was on brain-derived vesicles. These vesicles have indicated that the content of Aβ_42_ and various types of tau and, to a minor degree, synaptic and lysosomal proteins may be of use as biomarkers (for references see Refs.^16,17^). Although an elegant solution to isolate a specific subpopulation of EVs through immunoaffinity-derived methods, extra steps in the isolation procedure are warranted, which concomitantly result in a potential loss of relevant EVs. The specificity of the surface marker used to capture brain-derived EVs, i.e. L1CAM has been questioned due to its implications in cancer metastasis and presence in the tissue of the kidneys^18,19^. In addition, detection of proteins in a minute amount of EVs can be technically demanding. Furthermore, AD is a multifactorial disease, where the peripheral immune system and platelets have been previously implicated in the disease pathology^20,21^. Therefore, we have used a different approach with a simple and standardized procedure using ultracentrifugation of plasma to achieve differential isolation of all types of EVs.

The present study aimed at investigating the potential differences in protein content and relative abundance of blood-derived EVs from patients with AD and Mild Cognitive Impairment (MCI) compared to healthy controls and at elucidating disease-related EV proteome changes and its diagnostic potential.

Using a shotgun-based MS approach and feature selection statistics, we identified a subset of proteins distinguishing healthy from diseased individuals. These proteins were found to primarily be part of immunological and coagulation pathways. Some of the AD patients presented with highly elevated levels of coagulation factor XIII A1 (*FXIIIA1*) and B (*FXIIIB*). Additionally, we identified orosomucoid 2 (*ORM2*), retinol-binding protein 4 (*RBP4*), and hydrocephalus-inducing protein homolog (*HYDIN*) to be potential biomarkers for AD.

## Materials and methods

### Study participants

In total, 30 participants were included; 10 patients with AD, 10 patients with MCI, 10 healthy controls. Technical issues caused one control sample to not be analysed by MS. Patients were clinically verified at the Department of Neurology at Aalborg University Hospital and consecutively enrolled at the time of diagnosis, but before the initiation of treatment. For patients with mild to moderate AD, the diagnosis was based on the National Institute of Neurological and Communicative Disorders and Stroke and the Alzheimer’s Disease and Related Disorders Association (NINCDS-ADRDA)^22^ and International Classification of Diseases and Related Health Problems 10^th^ Edition (ICD_10_) criteria^23^. For MCI patients the diagnosis was based on the Petersen criteria^24^. When found necessary by the physician, patients’ cognition was tested using the mini-mental state examination (MMSE), Addenbrooke’s Cognitive Examination (ACE), Functional Activities Questionaire (FAQ), as well as paraclinical measurements of CSF markers Aβ, phospho-tau (p-tau), and total-tau (t-tau).

Age- and gender-related healthy donors from the blood bank at Aalborg University Hospital were included for comparison. Donors > 65 years completed a questionnaire related to their mental state, such as memory impairment prior to inclusion. All study participants signed a written informed consent form. The study was approved by the North Denmark Region Committee on Health Research Ethics (N-20150010) and conducted according to the Declaration of Helsinki.

### Sample collection and processing

Plasma samples from patients and healthy controls were collected and processed as previously described^25^. Briefly, blood was obtained from the median cubital vein using a 21-gauge needle (Vacuette, Greiner Bio-One, Austria). 9 mL 0.105 M (3.2%) trisodium citrate tubes were used and processed within 2 h after blood collection. Platelet-free plasma was obtained using centrifugation twice at 2500 × *g* at room temperature for 15 min. After each centrifugation, plasma supernatant was collected until 1 cm above the buffy coat. Samples were subsequently snap-frozen in liquid nitrogen and stored at − 80 °C until analysed.

### Biochemical analysis

Several biochemical analyses were performed to ensure no co-morbidities of the participants; alanine transaminase, albumin, carbamide, cholesterol, creatinine, C-reactive protein (CRP), glucose, high and low-density lipoprotein cholesterol, haemoglobin, lactate dehydrogenase, and triglycerides were performed as previously described^25^. FXIII and ORM were further investigated in plasma. Measurements of FXIII antigen levels (HemosIL, Bedford, MA, USA) and activity (Berichrom FXIII, Siemens Healthineers, Erlangen, Germany) in plasma were performed using the ACL TOP500 CTS (Instrumentation Laboratory, Bedford, MA, USA) and the Sysmex CS-2100i (Sysmex Europe GmbH, Norderstedt, Germany), respectively. Plasma ORM levels were measured using the Cobas 8000 Modular Analyzer (Roche Applied Science, Penzberg, Germany).

### Extracellular vesicle enrichment

EV enrichment was performed from 1 mL plasma with double centrifugation at 100,000 × *g*, 1 h, 4 °C using an Avanti J-30i centrifuge with a J A-30.50 fixed angle rotor, k-factor 280 (Beckman Coulter, Brea, CA, USA). After initial centrifugation, EVs were washed in 1 mL 0.22 µm filtered phosphate-buffered saline (PBS). The EV enriched pellet was resuspended in 20 µL filtered PBS for mass spectrometry and in 100 µL for EV characterisation.

### Extracellular vesicle characterisation

EV pellets were characterised using nanoparticle tracking analysis (NTA), western blotting, and transmission electron microscopy (TEM) with immunogold labelling (IEM). The methods describing these analyses and original uncropped images can be found in Supplementary File S1 and Supplementary Fig. S1, respectively.

#### Preparation by S-Trap micro spin columns

For digestion of proteins in EV pellets, the commercially available S-Trap Micro Spin Columns (Protifi, NY, USA) were used according to the manufacturer’s instructions. Briefly, EV pellets were lysed in solubilisation buffer (5% SDS, 50 mM triethylammonium bicarbonate (TEAB), pH 7.55). Proteins were reduced by adding Tris (2-carboxyethyl)phosphine hydrochloride (TCEP) to a final concentration of 10 mM and heated at 95 °C for 10 min, cooled to room temperature, and alkylated using 40 mM iodoacetamide (final concentration) in dark for 30 min. A final concentration of 1.2% phosphoric acid was added to the samples, followed by six times the volume of S-Trap binding buffer (90% MeOH, 100 mM TEAB, pH 7.1). Samples were loaded onto S-Trap spin columns, centrifuged at 4000 × *g* until all buffer had passed through. The trapped proteins were collected and washed thrice with S-Trap buffer at the same settings. A total of 20 µL digestion buffer (50 mM TEAB) was mixed with 2–5 µg trypsin and incubated overnight at 37 °C. Peptides were eluted using three stepwise buffers; first 50 mM TEAB (40 µL), followed by 0.2 % aqueous formic acid (40 µL), and finally by 50 % acetonitrile with 0.2 % formic acid (35 µL). After the addition of each buffer, samples were centrifuged at 4000 × *g*. The elutions were pooled, dried by vacuum centrifugation, and resuspended in buffer A (99.9 % water, 0.1 % formic acid). Peptide concentrations were measured by fluorescence using an EnSpire microplate reader (Perkin Elmer, Waltham, MA, USA) and diluted at 1 µg/µL.

### Label-free quantitative nano liquid chromatography–tandem mass spectrometry analysis

Liquid chromatography–tandem mass spectrometry was performed as previously described^26^ using the universal method on an Orbitrap Fusion Tribrid mass spectrometry platform from Thermo Scientific (Waltham, MA, USA). Internal mass calibration was performed by activating the EASY-IC using fluoranthene. Peptides were trapped on a µ-Precolumn (300 µm × 5 mm, C18 PepMap100, 5 µm, 100 Å, Thermo Scientific) and separated on an analytical column (EASY-Spray Column, 50 mm × 75 µm, PepMap RSCL, C18, 2 mm, 100 Å, Thermo Scientific). A 91 min. elution gradient was constructed by mixing buffer A with buffer B (99.9 % acetonitrile, 0.1 % formic acid). Initially, 2 % B was used increasing to 14 % at 3 min, 25 % at 34 min, 40 % at 36 min, 80 % at 37 min, 80 % at 53 min, 2 % at 54 min which was kept at 2 % until 91 min. Samples were injected twice with an amount of 1 µg of sample protein per injection in duplicates, except for one sample which was injected only once due to technical troubleshooting. Replicates were injected with an intermission of several hours to days. The acquisition was performed in the first 60 min of the gradient as previously described^26^ with full Orbitrap scans (375 – 1500 *m/z*), a resolution of 120,000, and an automatic gain control (AGC) target of 4 × 10^5^ with a maximum injection time of 50 ms. Each cycle time lasted 3 s. Precursor ions with the highest intensity were selected, with an intensity threshold set at 5 × 10^3^, and charge states 2 – 7 included. The linear ion trap was used for MS^2^ scans at a rapid scan rate with a collision-induced dissociation energy at 35 % and an AGC target of 2 × 10^3^ with a maximum injection time of 300 ms. Using the quadrupole, precursor ions were isolated using an isolation window of 1.6 *m/z*, with dynamic exclusion set to 60 s. The median technical coefficient of variation was calculated for the proteins in each sample. The mean of this was 13.3 %.

### Database searches

The 57 raw data files were searched against the human Uniprot database (downloaded 12/03/2019) and using MaxQuant version 1.6.5.0 (Max Planck Institute of Biochemistry, Martinsried, Germany) for label-free quantification (LFQ) analysis^27^. Carbamidomethyl (C) was used as fixed modification. The false discovery rate (FDR) for peptide-spectrum matches (PSMs), proteins, and sites were each set at 1 %. The minimum ratio count for LFQ was set to 1. Tandem MS (MS/MS) was required for LFQ comparisons. For quantification of proteins, unique and razor peptides, unmodified and modified with oxidation (M) or acetyl (protein N-terminal) were used. The function match between runs was used. Reverse sequences were used for decoy search and contaminant sequences were included in the search.

### Statistical analysis

Demographics and clinical characteristics were presented as mean with standard deviations (mean ± SD). Group differences were investigated using either Student’s *t*-test for MMSE, ACE, FAQ, CSF Aβ, CSF p-tau, and CSF t-tau or analysis of variance (ANOVA) for age, particle concentration, and particle mean size. Proteins were filtered for potential contaminants, reverse sequences, proteins only identified by site, and at least 2 unique peptides. LFQ values were log_2_ transformed and technical replicates were averaged. Distributions were assessed through histograms. Proteins had to have 70 % valid values in at least one group. Venn diagrams were used to investigate proteins common and unique for each group and matched to the top 100 proteins found in EV-related studies using the EV databases; Vesiclepedia^28^ and ExoCarta^29^ (downloaded 07/04/2020). Unsupervised principal component analysis (PCA) was used to assess data trends. A missing value imputation of width 0.3 and downshift 1.8 was used prior to PCA.

Differentially expressed proteins were identified between healthy individuals and AD patients using Student’s *t*-test. A *p* < 0.05 and log_2_ fold change (FC) > 1 or < − 1 was considered statistically significant. A permutation-based FDR < 0.05 with 250 randomizations was adapted to correct for multiple hypothesis testing, reported as *q*-values. Protein comparisons were depicted using non-log_2_ transformed LFQ values. Significant proteins were subjected to enrichment analysis and annotated with the top five significant gene ontology biological process (GOBP) terms using functional annotation clustering analysis by DAVID version 6.8^30,31^. Enrichment scores (ES) and Benjamini–Hochberg corrected *p*-values, *q*-values, were shown. STRING analysis was performed using the StringApp in Cytoscape version 3.8.2^32^ for protein–protein interactions relating to biological functions for the differentially expressed proteins, requiring a minimum interaction score of 0.4 (medium interaction). When more than one protein ID was listed, the first was used as the allocated protein. Seven of the proteins could not be identified.

The Random Forest algorithm in MetaboAnalyst 4.0 (Xia Lab, Quebec, Canada)^33^ was used to select proteins distinguishing AD patients and controls, with missing value imputation as described above. Models selected by Random Forest and biomarker candidates were presented as receiver operating characteristic (ROC) curves.

Perseus version 1.6.10.50 (Max Planck Institute of Biochemistry, Martinsried, Germany)^34^, IBM SPSS Statistics 26 (SPSS, Chicago, IL, USA), and GraphPad Prism 9.0.0 (GraphPad Software, La Jolla, CA, USA) were used. MaxQuant data and sample ID list can be found in Supplementary Table S1 and Supplementary File S2, respectively.

## Results

### Clinical characteristics of study populations

For the present study, the protein profiles measured by MS from enriched plasma-derived EVs were compared between the three groups; AD, MCI, and healthy controls. The clinical characteristics and biochemical parameters have been presented in the study by Nielsen et al*.*^25^. Briefly, measurements of biochemical parameters were within the reference intervals for all participants, except with few anomalies in individuals who had elevated triglyceride and LDL cholesterol levels, though not group-specific. These parameters included markers linked to organ functions, such as liver, kidney, acute-phase reactants, and haematological panels. For clinical measurements, MCI and AD patients presented with lowered MMSE and ACE scores, higher FAQ scores, and slightly lowered CSF Aβ and higher CSF p-tau and t-tau levels (Table 1). Although the age span of diseased and healthy individuals was overlapping, the difference was statistically significant. Furthermore, AD patients presented with significantly lower MMSE (*p*: 0.041) and ACE (*p*: 0.007) scores compared to the MCI patients.

**Table 1:** Demographics and clinical information of study populations. All values are presented as mean ± standard deviation. *Con* healthy controls, *MCI* mild cognitive impairment, *AD* Alzheimer’s disease, *MMSE* Mini-Mental State Examination, *FAQ* Functional Activities Questionnaire, *ACE* Addenbrooke’s Cognitive Examination, *CSF* cerebrospinal fluid, *Aβ* Amyloid-β, *p-tau* phospho-tau, *t-tau* total-tau. *Interval for 51–70 years of age. For 71–90 years of age the interval is < 500.

	Con (*n* = 9)	MCI (*n* = 10)	AD (*n* = 10)	*p*-value	Reference interval
**Demographics**					
Age (years)	65 ± 1.0	72 ± 5.0	70 ± 5.0	0.005	–
Male/female (*n*)	4/5	2/8	4/6	–	–
**Clinical characteristics**					
MMSE	–	27.4 ± 2.3	23.6 ± 4.6	0.041	–
FAQ	–	4.0 ± 2.0 (*n* = 3)	10.4 ± 4.6 (*n* = 5)	0.066	–
ACE	–	85.0 ± 5.6 (*n* = 6)	58.7 ± 16.5 (*n* = 3)	0.007	–
CSF Aβ	–	998.5 ± 482.6 (*n* = 4)	626.3 ± 260.9 (*n* = 6)	0.148	> 500
CSF p-tau	–	98.0 ± 61.3 (*n* = 4)	80.5 ± 29.5 (*n* = 6)	0.556	< 61
CSF t-tau	–	563.0 ± 363.9 (*n* = 4)	628.2 ± 288.9 (*n* = 6)	0.760	< 450*

### Characterisation of extracellular vesicles

**Figure 1:**
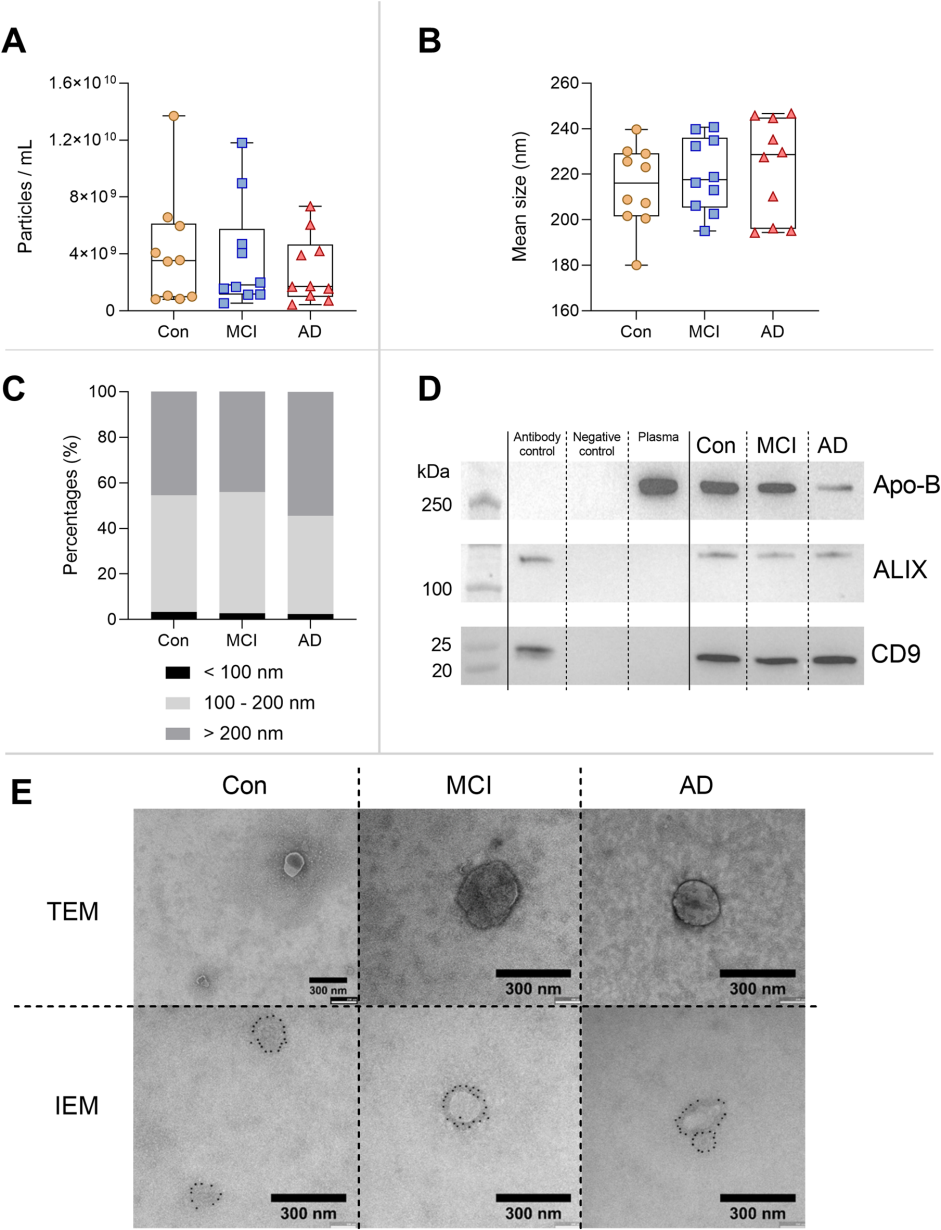
Characterisation of extracellular vesicles. NTA measurements show the particle concentrations (**A**) and mean particle sizes (**B**) for all three groups. (**C**) Size distribution of measured particles presented with similar profiles, with most particles in the size range of 100 – 200 nm. Boxplots depict median with interquartile ranges. (**D**) Western blotting of pooled EV pellets for all groups, confirming the presence of EV markers CD9 and ALIX, and the lipoprotein marker Apo-B. (**E**) TEM images of negative stains of pooled EV pellets from the three groups. IEM images present with CD9^+^ vesicular structures, with immunoreactivity confined to the outer membrane of the structures. The scale bars are 300 nm.

Results from NTA indicated no significant difference in the concentrations of measured particles in the EV pellets between the three groups (Fig. 1A). Similar observations were found for the mean size of the measured particles (Fig. 1B). The size distribution had similar profiles in all three groups (Fig. 1C). Most of the measured particles were within the size range of 100 – 200 nm; however, larger particles above 200 nm were also measured in these samples. Western blot confirmed the presence of the EV tetraspanin CD9, as well as the cytosolic marker ALIX (Fig. 1D). Co-isolation of contaminating lipoproteins was confirmed to be present in the samples using Apo-B (Fig. 1D), but was clearly reduced compared to plasma. Using both TEM and IEM, vesicular structures in the size range of 200 nm were observed in the samples, which were further confirmed to be CD9^+^ (Fig. 1E).

### Proteomic analysis of extracellular vesicle related proteins

**Figure 2:**
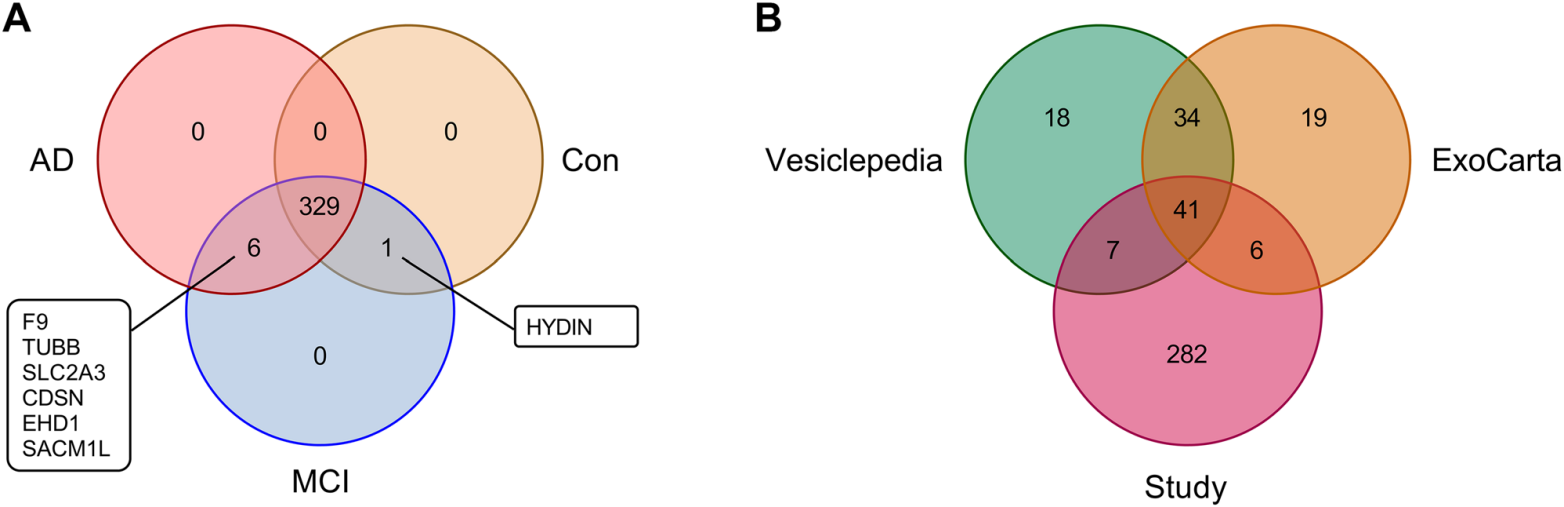
Visual representations of common and unique identified proteins amongst study groups and the EV databases based on Venn diagrams. (**A**) A total of 336 proteins are identified, with 329 common proteins for all groups, one protein common between healthy controls and MCI patients, and six proteins common between AD and MCI patients. No proteins are uniquely expressed in a single group. (**B**) With 336 proteins identified in this study, a total of 54 proteins overlap with top 100 proteins from the EV databases Vesiclepedia and ExoCarta, with 41 proteins shared between all three lists.

A total of 336 proteins were identified. Among these, 329 proteins were common for all three groups. One protein (*HYDIN*) was present in both healthy controls and MCI patients, however absent in the AD patient group. Six proteins (*F9*, *TUBB*, *SLC2A3*, *CDSN*, *EHD1*, and *SACM1L*) were present in both AD and MCI patients, but not in the healthy controls (Fig. 2A). None of the three groups contained uniquely expressed proteins. Furthermore, using the top 100 proteins associated with studies investigating EVs (Supplementary Table S2), based on the databases Vesiclepedia and ExoCarta, we investigated the number of proteins documented in these databases was also present in this study (Fig. 2B). Of the 336 proteins from the current study, a total of 54 proteins overlapped with the top 100 EV proteins, indicating that 16.1 % of the identified proteins were highly associated with EVs, although this observation does not indicate sample purity.

**Figure 3:**
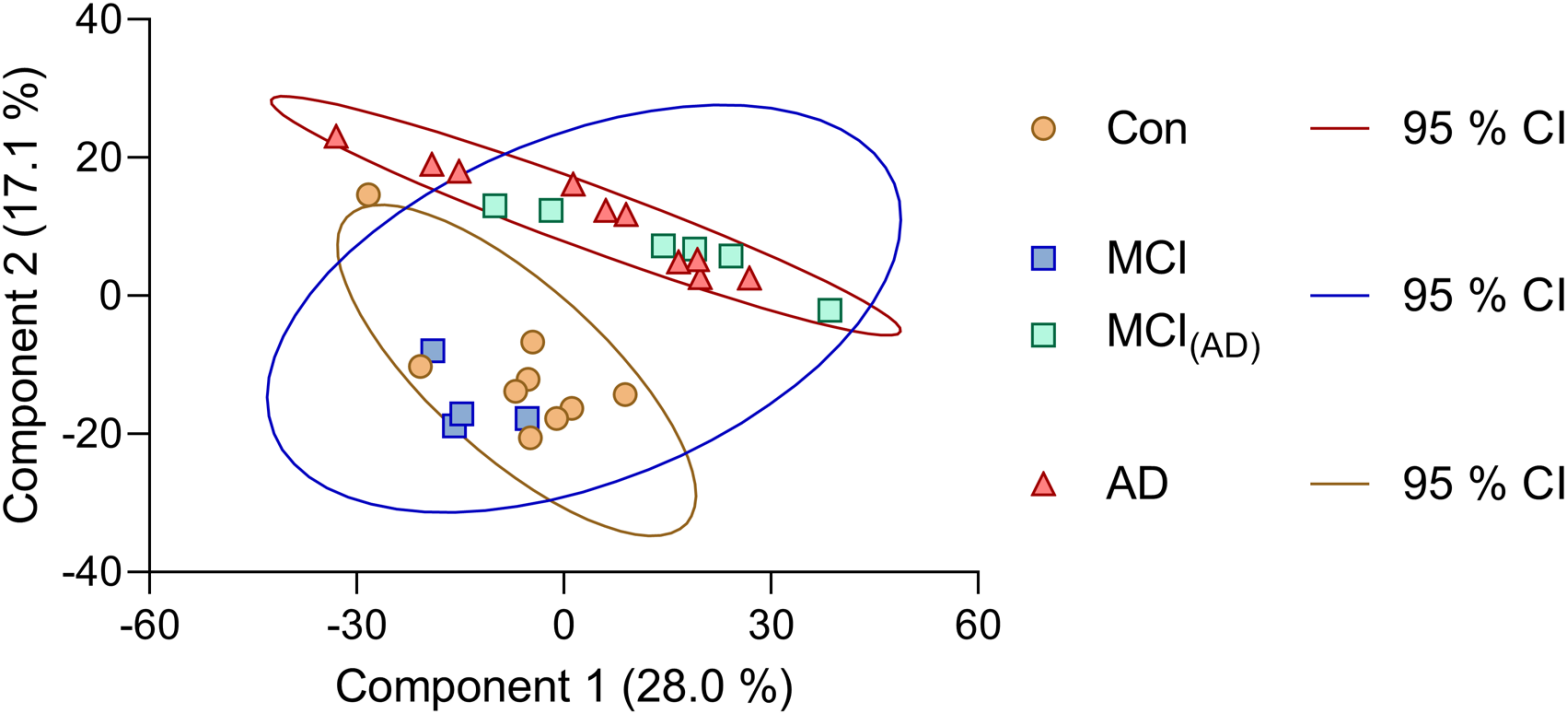
Scores plot with principal component analysis (PCA) results reveals samples clustering according to their groups; AD patients (red triangles), healthy controls (orange circles), and MCI patients. MCI patients cluster along with both the AD group (green squares, MCI_(AD)_) and control group (blue squares) indicating similarities with both groups. The corresponding 95 % confidence interval (CI) for the groups is indicated by the ellipses.

The protein profiles were further investigated for intra- and inter-group variations. PCA revealed clustering along the first and second principal components indicating differences in the protein profiles of subjects diagnosed with AD and healthy controls (Fig. 3). Interestingly, samples from the MCI group clustered with both the control (MCI) and AD groups (MCI_(AD)_), suggesting some similarities in these patient’s protein profiles that were related to either of the groups.

When filtering for 100 % valid values, to avoid skewness of data due to imputation, similar observations were made between the groups; however, the distance between the group clusters was less prominent compared to the PCA after 70 % filtration (Supplementary Fig. S2).

Seven of the 10 MCI patients have progressed to AD during the two years from blood sampling until now.

### Altered protein expression and pathways related to cognitive impairment

Based on the heterogeneity within the MCI patients as observed in the PCA plot, further comparisons were only focused between AD patients and healthy controls.

**Figure 4:**
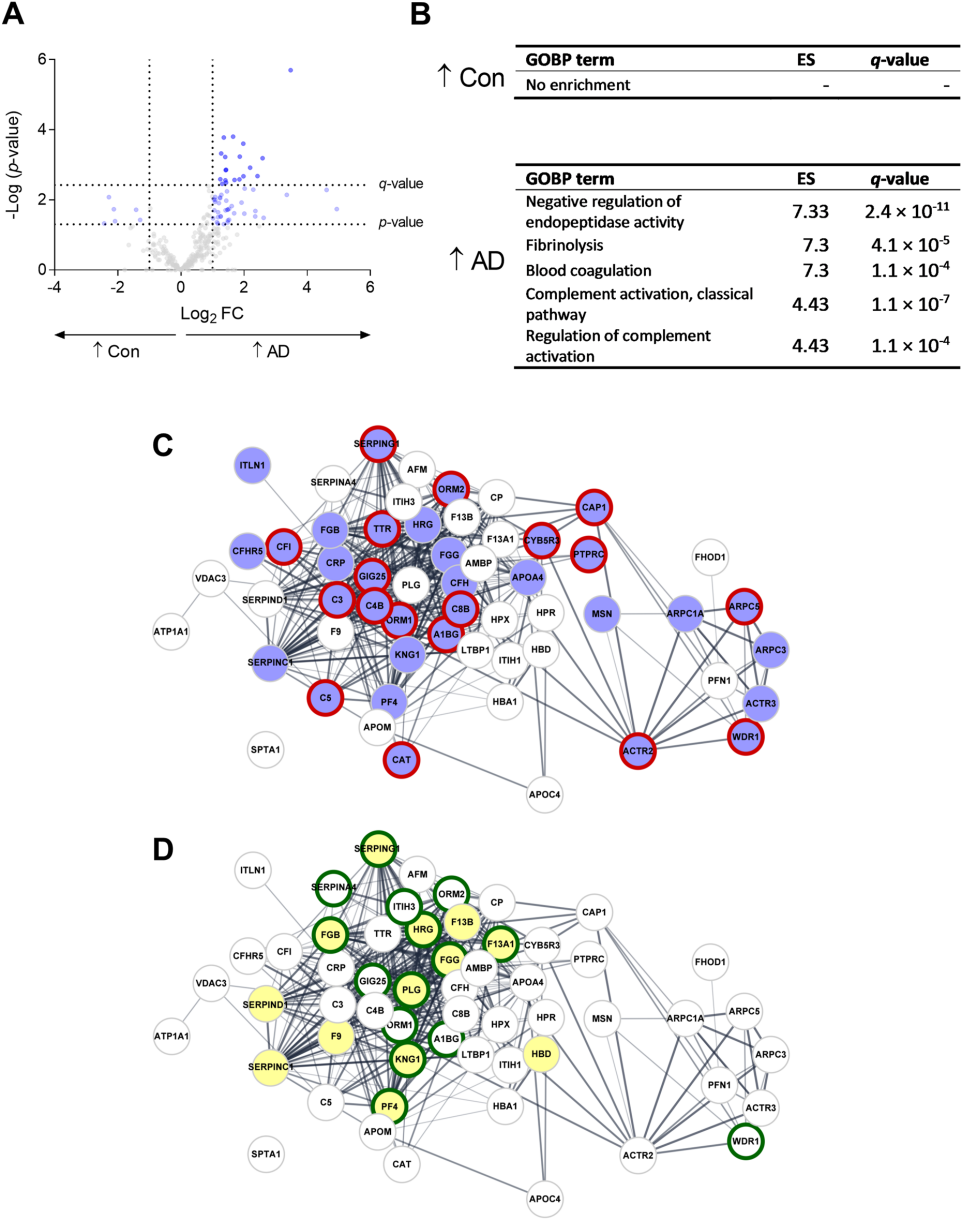
Significantly expressed proteins comparing AD patients and healthy individuals. (**A**) Volcano plot comparing AD patients with controls showed 57 proteins up-regulated in the AD group with 19 of them having a significant *q*-value and six proteins up-regulated in the control group. A cut-off value of a log_2_ FC > 1 or < − 1 is indicated, as well as *p*-value (light blue) and *q*-value (dark blue) < 0.05 cut-off lines. (**B**) Identified significant gene ontology terms for top five biological processes (GOBP), together with their enrichment scores (ES) and *q*-values are indicated. Downregulated proteins in AD patients show no enrichment of GOBP terms, while upregulated proteins show five terms from three different clusters. (**C**) Protein–protein interaction network analysis of 56 up-regulated proteins in AD. The nodes represent the proteins and the edges show their interactions. A significantly higher number of edges (334) is identified compared to the expected (35). Enrichment analysis reveals that these proteins are part of the immune system process (blue nodes) and leukocyte mediated immunity (red borders), and (**D**) platelet degranulation (green borders) and blood coagulation (yellow nodes).

Analysis of protein expressions between AD patients and healthy controls revealed 63 proteins being differentially expressed, with 57 upregulated in the AD group, and 19 of these being statistically significant after FDR correction (Table 2 and Fig. 4A). Proteins upregulated in the AD group indicated GOBP terms related to the inflammatory response (complement activation, classical pathway and regulation of complement activation) and coagulation processes (fibrinolysis and blood coagulation) (Fig. 4B). STRING analysis of upregulated proteins from AD patients, showed partially biologically related interactions. These proteins had 334 edges, which is significantly more than the 35 expected edges, meaning that the proteins have more interactions among themselves compared to that of a random set of a similar number of proteins. Enrichment analysis revealed that the proteins were mainly involved in biological related to the immune system, leukocyte-mediated immunity, platelet degranulation, and blood coagulation (Fig. 4C,D).

**Table 2:** Significantly expressed proteins comparing AD patients with healthy controls. *Permutation-based FDR in Perseus indicated a very low *q*-value approximated to 0. Benjamini–Hochberg indicated a *q*-value of 0.0006. The reported *q*-value is estimated to be below 0.0006.

AD│Con					
Uniprot ID	Gene name	Protein name	Log_2_ FC	*p*-value	*q*-value
P19652	ORM2	Orosomucoid 2	3.5	0.000002	< 0.0006*
P04433	IGKV3D-11	Ig kappa chain V–III region VG	2.0	0.0002	0.012
P19827	ITIH1	Inter-alpha-trypsin inhibitor heavy chain H1	1.4	0.0002	0.013
P04217	A1BG	Alpha-1B-glycoprotein	2.6	0.0007	0.017
P02042	HBD	Hemoglobin subunit delta	1.4	0.0006	0.018
O95445	APOM	Apolipoprotein M	1.7	0.0002	0.020
P05155	SERPING1	Plasma protease C1 inhibitor	1.9	0.0006	0.021
P02760	AMBP	Alpha-1-microglobulin	1.3	0.0005	0.022
P05546	SERPIND1	Heparin cofactor 2	1.4	0.001	0.029
P01008	SERPINC1	Antithrombin-III	2.2	0.001	0.030
P05023; P13637	ATP1A1; ATP1A3	Na^+^/K^+^-transporting ATPase subunit alpha-1; Na^+^/K^+^-transporting ATPase subunit alpha-3	1.4	0.001	0.031
P07737	PFN1	Profilin-1	2.0	0.002	0.036
P00747	PLG	Plasminogen	2.4	0.002	0.039
Q01518	CAP1	Adenylyl cyclase-associated protein 1	1.4	0.003	0.039
P01860	IGHG3	Ig gamma-3 chain C region	1.9	0.003	0.041
Q9BXR6	CFHR5	Complement factor H-related protein 5	1.7	0.003	0.041
P02763	ORM1	Orosomucoid 1	1.2	0.003	0.043
P02790	HPX	Hemopexin	1.4	0.003	0.044
P02776	PF4	Platelet factor 4	1.3	0.003	0.047
P01859	IGHG2	Ig gamma-2 chain C region	2.0	0.005	0.059
P61160	ACTR2	Actin-related protein 2	1.5	0.006	0.060
P00488	F13A1	Coagulation factor XIII A chain	4.6	0.005	0.060
P00739	HPR	Haptoglobin-related protein	2.3	0.005	0.061
P02549	SPTA1	Spectrin alpha chain, erythrocytic 1	3.4	0.007	0.070
P01011	SERPINA3	Alpha-1-antichymotrypsin	1.2	0.009	0.071
P00451	F8	Coagulation factor VIII	− 2.3	0.008	0.071
P02766	TTR	Transthyretin	1.2	0.009	0.072
P04196	HRG	Histidine-rich glycoprotein	1.3	0.007	0.072
P06727	APOA4	Apolipoprotein A-IV	1.1	0.008	0.073
P43652	AFM	Afamin	1.0	0.010	0.077
P55056	APOC4	Apolipoprotein C-IV	1.4	0.011	0.078
P01042	KNG1	Kininogen-1	1.1	0.010	0.078
P00450	CP	Ceruloplasmin	1.1	0.011	0.081
O75083	WDR1	WD repeat-containing protein 1	1.9	0.012	0.083
Q9Y277	VDAC3	Voltage-dependent anion-selective channel protein 3	1.2	0.012	0.083
P05156	CFI	Complement factor I	1.6	0.015	0.102
P04040	CAT	Catalase	1.4	0.020	0.108
P01031	C5	Complement C5	1.5	0.019	0.109
P08603	CFH	Complement factor H	1.1	0.020	0.110
P06312	IGKV4-1	Ig kappa chain V–IV region	2.4	0.021	0.110
P62987; P62979; P0CG47;P0CG48	UBA52; RPS27A; UBB; UBC	Ubiquitin-60S ribosomal protein L40; Ubiquitin-40S ribosomal protein S27a; Polyubiquitin-B; Polyubiquitin-C	− 1.4	0.019	0.111
P05160	F13B	Coagulation factor XIII B chain	4.9	0.018	0.112
P0DOY3; P0DOY2	IGLC3; IGLC2	Immunoglobulin lambda constant 3; Immunoglobulin lambda constant 2	− 2.1	0.018	0.112
P26038	MSN	Moesin	1.5	0.019	0.113
P0C0L5	C4B	Complement C4-B	1.5	0.018	0.113
P00387	CYB5R3	NADH-cytochrome b5 reductase 3	1.2	0.023	0.118
P02675	FGB	Fibrinogen beta chain	1.7	0.024	0.125
Q8WWA0	ITLN1	Intelectin-1	2.0	0.025	0.127
P29622	SERPINA4	Kallistatin	1.3	0.030	0.138
P07358	C8B	Complement component C8 beta chain	1.3	0.030	0.140
P02741	CRP	C-reactive protein	2.4	0.030	0.142
P61158	ACTR3	Actin-related protein 3	1.2	0.031	0.143
Q9Y613	FHOD1	FH1/FH2 domain-containing protein 1	2.6	0.032	0.144
P69905	HBA1	Hemoglobin subunit alpha	1.0	0.034	0.146
P30740	SERPINB1	Leukocyte elastase inhibitor	− 1.3	0.037	0.155
P02679	FGG	Fibrinogen gamma chain	1.5	0.037	0.156
A0A0J9YX35	IGHV3-64D	Immunoglobulin heavy variable 3-64D	1.5	0.039	0.159
P47929	LGALS7	Galectin-7	− 2.1	0.041	0.165
A0A0B4J1V1; P01762	IGHV3-21	Ig heavy chain V–III region TRO	1.1	0.043	0.174
Q14766	LTBP1	Latent-transforming growth factor beta-binding protein 1	1.5	0.044	0.174
Q01469	FABP5	Fatty acid-binding protein, epidermal	− 2.4	0.047	0.181
Q06033	ITIH3	Inter-alpha-trypsin inhibitor heavy chain H3	1.2	0.048	0.182
P08575	PTPRC	Receptor-type tyrosine-protein phosphatase C	1.4	0.049	0.183

### Biomarker candidates for Alzheimer’s disease

To identify patterns of regulated proteins enabling classification for diagnosis of cognitive impairment, feature selection using Random Forest was employed.

**Figure 5:**
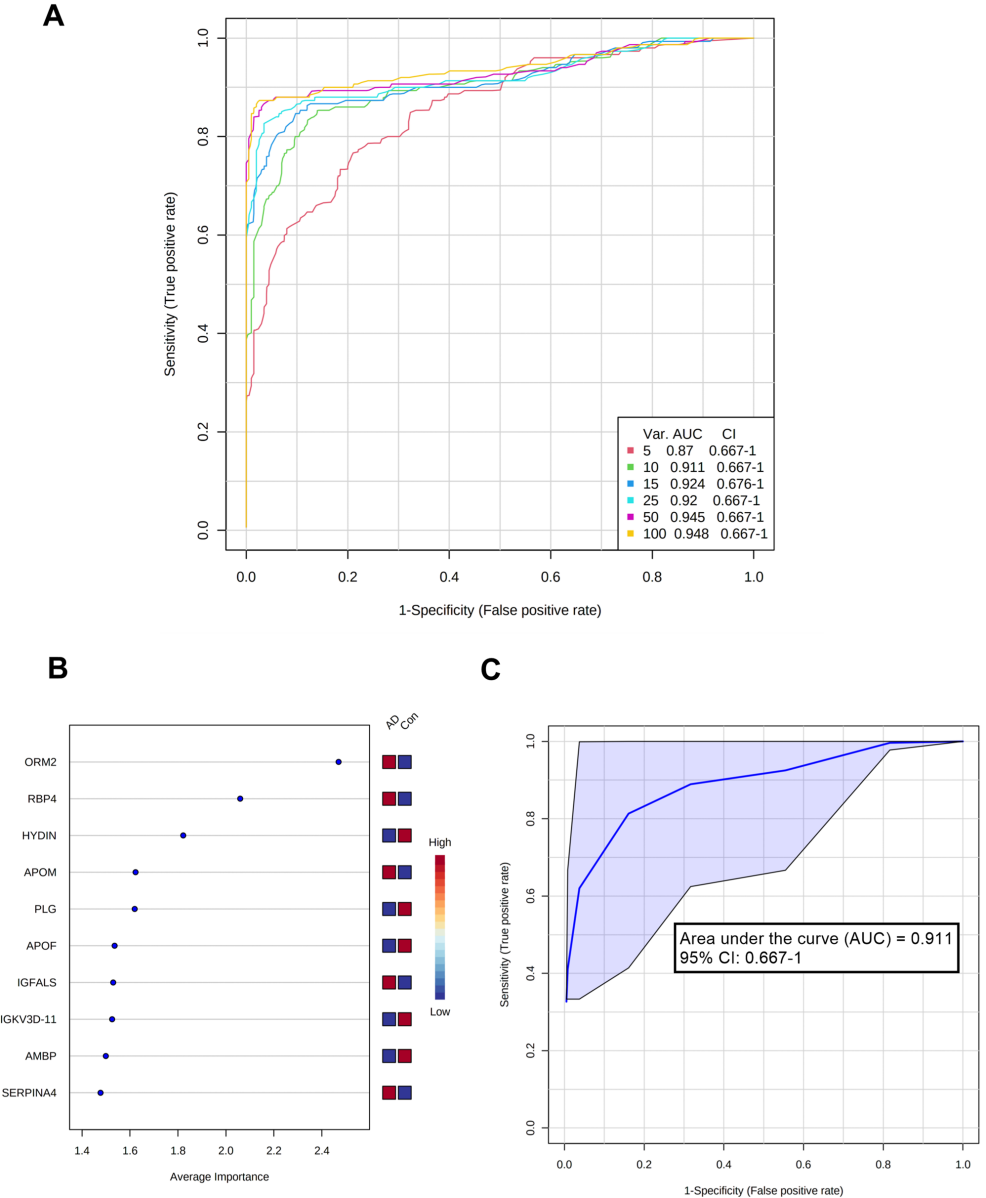
Random Forest analysis of important proteins for candidate biomarker models for AD diagnostics. (**A**) Receiver operating characteristic (ROC) curves of six models with increasing number of proteins (5, 10, 15, 25, 50, and 100 proteins). (**B**) Top 10 important features (proteins) based on average importance for model building. The intensity bar indicates the relevance of the specific protein for the AD or control group. (**C**) ROC curve for the model using the top 10 proteins to distinguish between cognitively affected and healthy individuals.

An increasing number of proteins were used to create discriminating models for AD patients and healthy controls containing 5, 10, 15, 25, 50, and 100 proteins (Fig. 5A). The 100 proteins used for these ROC curves can be found in Supplementary Table S3. The 10 most interesting discriminatory proteins consisted of *ORM2*, *RBP4*, *HYDIN*, *APOM*, *PLG*, *APOF*, *IGFALS*, *IGKV3D-11*, *AMBP*, and *SERPINA4* (Fig. 5B), with an overall performance of AUC: 0.91 and 95% CI: 0.67–1.00 (Fig. 5C).

Based on the ranked important proteins from the Random Forest analysis, three proteins showed a noticeably higher average importance (*ORM2*, *RBP4*, and *HYDIN*). Furthermore, two proteins were of special interest when comparing the protein profiles from AD patients to that of healthy controls. Both coagulation factor XIII subunits, *FXIIIA1* and *FXIIIB*, presented with the highest log_2_ FC (*FXIIIA1* log_2_ FC: 4.6 and *FXIIIB* log_2_ FC: 4.9). Therefore, these proteins were further investigated for their properties as possible single putative biomarker candidates.

**Figure 6:**
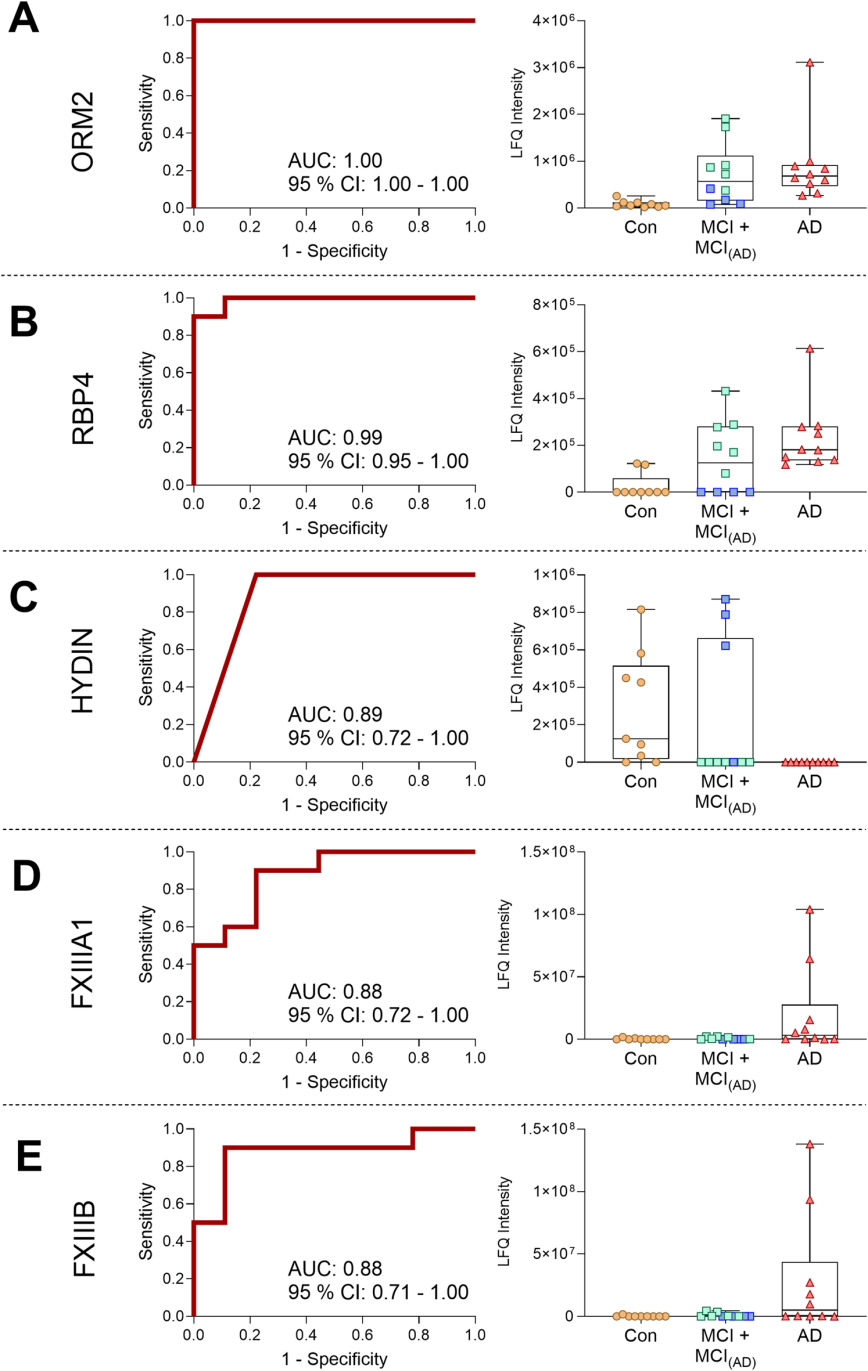
Receiver operating characteristic (ROC) curves and their corresponding boxplots of protein biomarker candidates for AD. (**A**) Orosomucoid 2 (*ORM2*). (**B**) Retinol binding protein 4 (*RBP4*). (**C**) Hydrocephalus-inducing protein homolog (*HYDIN*). (**D**) Coagulation factor 13 A1 (*FXIIIA1*). (**E**) Coagulation factor 13 B (*FXIIIB*). Boxplots show non-logarithmic label free quantification (LFQ) intensities and include NaN values arbitrarily set to 0. Both subpopulations of the MCI group are indicated in the boxplots with MCI_(AD)_ group marked with green squares.

*ORM2* and *RBP4* showed excellent ROC curves with an AUC of 1.00 (95% CI: 1.00–1.00) and AUC of 0.99 (95% CI: 0.95–1.00), respectively (Fig. 6A,B). The protein *HYDIN* presented with a slightly lower AUC of 0.89 (95% CI: 0.72–1.00) (Fig. 6C). This protein was below the detection limit for the AD group, in contrast to the two former proteins, which were observed to be highly upregulated compared to the control group. Interestingly, as observed in the PCA plot (Fig. 3), six of the MCI patients (MCI_(AD)_) clustered with the AD group, and the proteins from the MCI_(AD)_ patients were similarly expressed as the AD patients (Fig. 6A,C). Secondly, the two coagulation factor XIII subunits, *FXIIIA1* and *FXIIIB*, although with the highest log_2_ FC, presented with ROC curves with an AUC of 0.88 (95% CI: 0.72–1.00) for *FXIIIA1* and an AUC of 0.88 (95% CI: 0.71–1.00) for *FXIIIB* (Fig. 6D,E). Two of these proteins (*RBP4* and *HYDIN*) were not found to be statistically significantly different in protein comparisons in Table 2, which is due to the missing value imputations used for the Random Forest analysis, since most of their values were below the detection limit in the control group for *RBP4* and the AD group for *HYDIN*.

Due to the observed differences of proteins in EV samples, we investigated if such changes could also be observed in plasma. Therefore, levels of ORM2 were measured in plasma samples, together with levels and activity of FXIII. Interestingly, comparisons of these protein levels and activity in plasma samples did not show any statistically significant differences amongst the groups (Supplementary Fig. S3), which is in contrast to our findings of relative protein expressions in EV samples.

The results presented in this study further corroborates the importance of investigating EVs and their protein cargo for biological information related to AD pathogenesis and cognitive impairment, as well as relevant biomarker candidates for the diagnosis of AD.

## Discussion

The present study investigated the EV proteome derived from cognitively affected AD and MCI patients, and compared it to that of healthy individuals to identify potential biomarker candidates. Distinctive protein profiles were found to efficiently distinguish AD patients from healthy individuals. Ultracentrifugation was selected as the method of choice for our EV enrichment. This selection was based on criteria for easy implementation into a clinical setting, together with the high yield of EVs. Also, for studies of diagnostic character, a high yield is prioritized more than the purity of the sample material^35^, and ultracentrifugation fits these criteria.

Characteristics of EV enrichment revealed no significant differences in concentrations and size of the isolated particles in EV samples. This is in agreement with our previous observations, characterising EVs from a 20,000 × *g* centrifugation^25^. Another study has investigated plasma-derived EVs from AD patients and found a significant difference in particle concentrations and size compared to healthy individuals, with a lower particle concentration and larger particle size in AD patients^36^. Although our data did not show any significant differences, a similar trend could be observed, with a mean lower amount of particles in AD and larger particles measured. Enriched particles in the pellets were confirmed to contain EV specific markers CD9 and ALIX using western blot. CD9^+^ EVs were further confirmed by IEM. The identified proteins were compared to known EV databases, ExoCarta and Vesiclepedia, and several important EV related proteins were found present in our study. Based on a study conducted by Kowal et al*.*^37^, these EV related proteins could be divided into specific subgroups depending on EV properties; proteins in large EVs (*ACTN4* and *ACTN1*), proteins in light small EVs (*ADAM10*), and proteins in multiple EVs (*GAPDH* and *CD9*). Furthermore, the analysed samples also contained proteins associated with lipoproteins *APOA1*, *APOA2*, and *APOB*, which can be co-precipitated contaminants during EV enrichment^38^. Thus, proteins linked to EVs were identified, together with known contaminants, which was an expected outcome considering the nature of the EV enrichment method used in the study^39^.

Neuroinflammation is an integral part of the AD pathology, where both brain-resident immune cells and the peripheral innate immune response play a central role in pathological processes^40^. ORM, an acute-phase protein existing as two subtypes in humans, *ORM1* and *ORM2*, is mainly produced in the liver. *ORM1* accounts for approximately 75 % of the subtypes and the protein is sharply increased under pathological conditions, such as inflammatory stimuli^41^. In a mouse model, *ORM2* has been shown to be the predominant subtype present in brain tissue^41,42^. During late phase inflammation reactive astrocytes release *ORM2* to modulate anti-inflammatory activity on activated microglia with impaired clearance function due to continued Aβ stimulation^42^. The study also observed that only the hippocampal astrocytes appeared to produce *ORM2* during systemic inflammation, which is interesting, as the hippocampus is the most vulnerable brain region to an inflammatory response, as well as one of the first areas in the brain to be affected by AD pathology^42^. Moreover, the authors found significantly increased levels of *ORM2* in plasma from MCI and AD patients compared to healthy controls^42^. Their findings are somewhat in contrast to ours, as we only detected a difference in EV isolates and not in plasma samples of the AD patients. Similar observations have been made for CRP, where an increased expression was found in EVs; however, no differences were measured in serum CRP^25^. Tight junction proteins are important BBB components to maintain its functional integrity, and a previous study has shown that *ORM2* is able to positively affect the BBB integrity through increasing the expression of tight junction proteins zonula occludens 1 and occludin^43^. Our findings of increased *ORM2* expression could thus be a positive response to regulate the inflammatory responses and an attempt to re-establish BBB integrity.

In this study, we found *FXIIIA1* and *FXIIIB* to be highly elevated in some AD patients, and the literature shows an interesting connection between levels of FXIII and disease pathology. FXIII exists as a tetramer present in the circulatory system, consisting of two A subunits and two carrier B subunits. The A subunit is the catalytic active part with the transglutaminase function^44^. Aβ proteins accumulate along the brain vasculature in cerebral amyloid angiopathy (CAA), which is present in over 90 % of AD patients^45^. In a mouse model for chronic cerebral hypoperfusion, Shi et al*.*^46^ found significantly expressed FXIIIA in several brain areas such as the hippocampus, thalamus, and neocortex. These findings indicated an association between FXIII, BBB impairment, and cerebrovascular damage, further contributing to AD-related neurodegeneration. Due to the transglutaminase properties, FXIII has been shown to cross-link Aβ into highly stable multimers, which are resistant to proteolytic breakdown^47,48^. These FXIII-Aβ complexes were also shown to co-localize along the cerebral vasculature in CAA^48^. In addition, de Jager et al*.*^48^ showed a protective function of these complex formations, where the binding of FXIII with Aβ protected smooth muscle cells in the cerebral vasculature from the cytotoxicity of Aβ, thus avoiding further damage to the BBB by the formation of a highly stable clot^48^. Lastly, FXIII has been positively associated with neuron-derived EVs released after traumatic brain injury. Furthermore, the FXIII protein was found to bind to neurotoxic forms of Aβ and by EVs delivering these cross-linked proteins into near and distant neurons^49^.

In our study, ORM and FXIII were found significantly elevated in EV enriched samples, but not in plasma. The amount of EVs was comparable among the groups, as indicated by the NTA measurements. These proteins (FXIII and ORM) could either be loaded into the EVs during biogenesis or bound to the surface of EVs, as plasma proteins are known to do^50^. Since the amount of EVs is comparable across all individuals, the increased amount observed could be related to a higher binding affinity of the proteins for the disease-related EVs, if the proteins are bound to these entities. Alternatively, an increased amount of these proteins is possibly loaded into the EVs in pathological conditions.

*RBP4* is secreted by adipocytes as an adipocyte-derived hormone, adipokine^51^. In addition, *RBP4* has been shown to bind transthyretin, a carrier protein able to transport Aβ from the brain to the periphery, thus resulting in lower Aβ neurotoxicity^52^. Furthermore, *RBP4* has also been shown to transport retinols, such as vitamin A^53^. Vitamin A has been shown to have anti-oxidative and cell-protective effects^54^ and be able to inhibit the formation of Aβ oligomers in AD^55^. Thus, the increased intensities observed in this study could potentially be a response to the increasing accumulation of Aβ in relation to the AD pathology. However, in the current literature there are conflicting findings related to *RBP4* in AD, probably due to the sample material analysed. In post-mortem brain samples, *RBP4* expression has been found increased in AD compared to healthy individuals^56^. Another study found gradually decreased levels of *RBP4* in CSF samples from controls to MCI patients and finally to severe cases of AD^57^. Ishii et al*.*^51^ sought to investigate peripheral levels of *RBP4* in plasma samples as a potential biomarker for AD; however, they found no differences between AD and cognitively healthy subjects. As we did not use plasma directly, but EVs derived from the circulation, this could be an explanation for the discrepancy. Further similar investigations are needed to determine the role of *RBP4* as a potential biomarker for AD.

The brain ventricles are covered with motile ciliated epithelium, aiding the distribution and flow of the CSF from the choroid plexus^58^. Cilia consists of an axoneme, a microtubule-based cytoskeletal structure, with *HYDIN* being a known axonemal protein^59^. Mutations are known to occur in the *HYDIN* gene, causing dysregulation of ciliary movement and leading to hydrocephalus, an excessive accumulation of CSF within ventricles of the brain^59^. Ventricular enlargement is a characteristic of neuropathological changes associated with cognitive impairments, including MCI and AD^60^. Our EV measurements of *HYDIN* indicated that it was not measurable in AD, which could be explained by atrophy of the brain tissue in AD, usually identified by magnetic resonance imaging^60^, thus causing dysregulation of the protein expression and ciliary movement, leading to ventricular enlargement. However, the role of *HYDIN* in relation to AD need to be further investigated before any concise role can be determined for its involvement in disease pathology.

Both STRING and DAVID enrichment analyses showed that proteins upregulated in AD were involved in biological processes related to immunological and coagulation processes. We have presented proteins involved in these processes, such as FXIIIA1, FXIIIB, and ORM2. Furthermore, leukocyte-mediated immunity and platelet degranulation have also been shown to play a role in AD pathology. There is evidence of involvement of the peripheral immune system in AD, with infiltrating leukocytes aiding in phagocytosis of Aβ. However, it is also proposed that these infiltrating cells change to an inflammatory phenotype, due to the neuroinflammation in the brain environment, and thus contributing to this ongoing inflammatory process^20^. Platelets are the main source (~ 90 %) of amyloid precursor protein (APP) and Aβ in blood. Aβ is stored in α-granules and released upon stimulation causing degranulation e.g. by thrombin, thus contributing to the level of circulating Aβ. Soluble APP can exert inhibition of platelet degranulation, while Aβ counteracts this effect^61^.

This study has some limitations. Firstly, small patient and control populations were used for this exploratory study, even though clear differences were observed between healthy individuals and patients with AD. Secondly, not all clinically verified AD and MCI patients had measurements of CSF Aβ and tau biomarkers, as it was not necessary for the diagnosis. These measurements of established biomarkers could have been correlated with our proposed biomarker candidates. Thirdly, the control group had a slightly lower age compared to the patient groups, as recruitment of older blood donors was not possible. This age difference was also significant, however, given that this age span is grouped in proteomics studies, this was probably of minor importance^62^. Fourthly, proteomics data contains missing values, which have been imputed for some of the analyses.

Thus, our findings elaborated on the global effort of identifying blood-based biomarkers for the diagnosis of AD. However, with a discovery-based study, further investigations are warranted to replicate our findings in a larger independent population of cases and controls. Seven of the patients that were initially classified as MCI progressed to AD during the study period. Some of these also revealed increased levels of *ORM2* and *RBP4,* indicating their potential as biomarkers for disease progression.

## Conclusions

Based on our initial work, the main finding is that EVs provide an accessible matrix for biomarker discovery, with several proteins involved in inflammation and coagulation processes. We found 10 proteins to distinguish AD patients from healthy individuals. Especially *ORM2*, *RBP4*, and *HYDIN* showed high specificity sensitivity. Furthermore, coagulation factor XIII subunits *FXIIIA1* and *FXIIIB* presented to be significantly upregulated in some of the AD patients. Some of the MCI patients presenting similar protein profiles as the AD patients progressed to clinically verified AD within two years, giving confidence to our findings.

## Supplementary Information

Supplementary Information 1.

Supplementary Information 2.

Supplementary Table S1.

Supplementary Table S2.

Supplementary Table S3.

## Data Availability

The mass spectrometry proteomics data have been deposited to the ProteomeXchange Consortium via the PRIDE^63^ partner repository with the dataset identifier PXD024216. In addition, we have submitted all relevant data of our experiments to the EV-TRACK knowledgebase (EV-TRACK ID: EV210181)^64^.
