# Supplementary material for: Shotgun-based proteomics of extracellular vesicles in Alzheimer’s disease reveals biomarkers involved in immunological and coagulation pathways
Source: Sci Rep. 2021 Sep 16;11:18518. doi: 10.1038/s41598-021-97969-y (PMC8445922; doi:10.1038/s41598-021-97969-y)

**Legends for Supplementary Tables, Figures, and Files**

**Supplementary Table S1.** MaxQuant mass spectrometry data file containing all raw data.

**Supplementary Table S2.** The top 100 proteins associated with studies of extracellular vesicles from the Vesiclepedia and ExoCarta databases.

**Supplementary Table S3.** The 100 proteins selected from the Random Forest feature selection model.

**Supplementary Figure S1.** Original and uncropped western blot and electron microscopy images.

**Supplementary Figure S2.** Principal component analysis (PCA) using 100 % valid values in all groups. The AD patient group (red triangles) and control group (orange circles) present with a small overlap of their 95 % confidence interval (CI). The MCI patient group is observed to cluster in both the AD group (green squares, MCI_(AD)_) and control group (blue squares). 95 % CIs are indicated by the ellipses.

**Supplementary Figure S3.** Plasma measurements of (**A**) Coagulation factor 13 (FXIII) antigen levels, (**B**) FXIII activity, and (**C**) Orosomucoid (ORM) concentrations. No statistically significant differences were observed between the groups for either of the measured proteins.

**Supplementary File S1.** Methods section describing the methods used for characterisation of extracellular vesicles. The methods include nanoparticle tracking analysis, western blotting, and transmission electron microscopy.

**Supplementary File S2.** An ID list for samples analysed by mass spectrometry.

**Supplementary File S1**

**Nanoparticle Tracking Analysis**

Nanoparticle tracking analysis was performed to measure the size and concentration of particles present in EV samples as previously described^24^. Briefly, a LM10-HS system with a red 638 nm laser (Malvern Instruments Ltd, Malvern, UK) coupled with a CCD camera (Allied Vision Technologies GmbH, Stadtroda, Germany) was used. System settings included camera level 11, threshold 3 with blur 9 × 9, and 5 video recordings or 30 seconds duration. Nanosight NTA software version 3.2 (Malvern Instruments Ltd, Malvern, UK) was used.

**Western Blotting**

Western blotting was performed to validate the presence of markers specific to EVs, as well as contaminating markers, as previously described^24^. Briefly, primary antibodies used included monoclonal mouse anti-CD9 antibody (clone M-L13, BD Pharmingen, San Diego, CA, USA), monoclonal mouse anti-apolipoprotein B (Apo-B, clone F2C9, Thermo Scientific, Waltham, MA, USA), and polyclonal rabbit anti-programmed cell death 6-interacting protein (ALIX, Merck Millipore, Burlington, MA, USA) diluted 1:500, 1:1000, and 1:1000, respectively. Secondary antibodies included horseradish peroxidase-conjugated polycloncal goat anti-mouse immunoglobulins/HRP and Amersham ECL donkey anti-rabbit IgG, HRP-linked F(ab’)_2_ fragment (GE Healthcare, Little Chalfont, UK), both diluted 1:30,000. A platelet lysate was included as a positive control for CD9 and ALIX and diluted plasma was used for Apo-B. PBS was used as a negative control.

**Transmission Electron Microscopy with Immunogold Labelling**

Transmission electron microscopy (TEM) was used to confirm the presence of structurally intact CD9^+^ vesicles in the EV pellets. TEM was performed as previously described^24^. Briefly, EV isolates were stained using 1 % (*w/v*) phosphotungstic acid (PTA), pH 7.0 (Ted Pella, Caspilor AB, Lindingö, Sweden) for 30 seconds on carbon-coated, glow discharged 400 mesh Ni grids (SPI Supplies, Chester, PA, USA). For phenotypical characterization using CD9, additional steps were included. Before staining, EV pellets were blocked using 0.5 % ovalbumin (Sigma-Aldrich, St. Louis, MO, USA), followed by primary antibody incubation for 30 minutes at 37 °C using monoclonal mouse anti-CD9 antibody (clone M-L13, BD Pharmingen, San Diego, CA, USA) 1:50 with 0.5 % ovalbumin in PBS. A secondary antibody used was 10 nm gold-conjugated goat anti-mouse (British BioCell, Cardiff, UK) 1:25 in 0.5 % ovalbumin in PBS. Then, three times 10 minutes incubation with 1 % cold fish gelatin (Sigma-Aldrich, St. Louis, MO, USA) was performed. Lastly, a staining procedure as mentioned above was performed. The grids were examined in a JEM-1400Flash electron microscope operated at 60 keV (JEOL, Tokyo, Japan) equipped with a TVIPS TemCam FX416 digital camera (TVIPS, Gauting, Germany). A scale bar was recorded on each image. ImageJ software version 1.51j8 (NIH, Bethesda, MD, USA) was used for editing.

**Supplementary Figures S1 – S3**

**Figure S1.** Original and uncropped western blot and electron microscopy images.

Western blot image – Apo-B


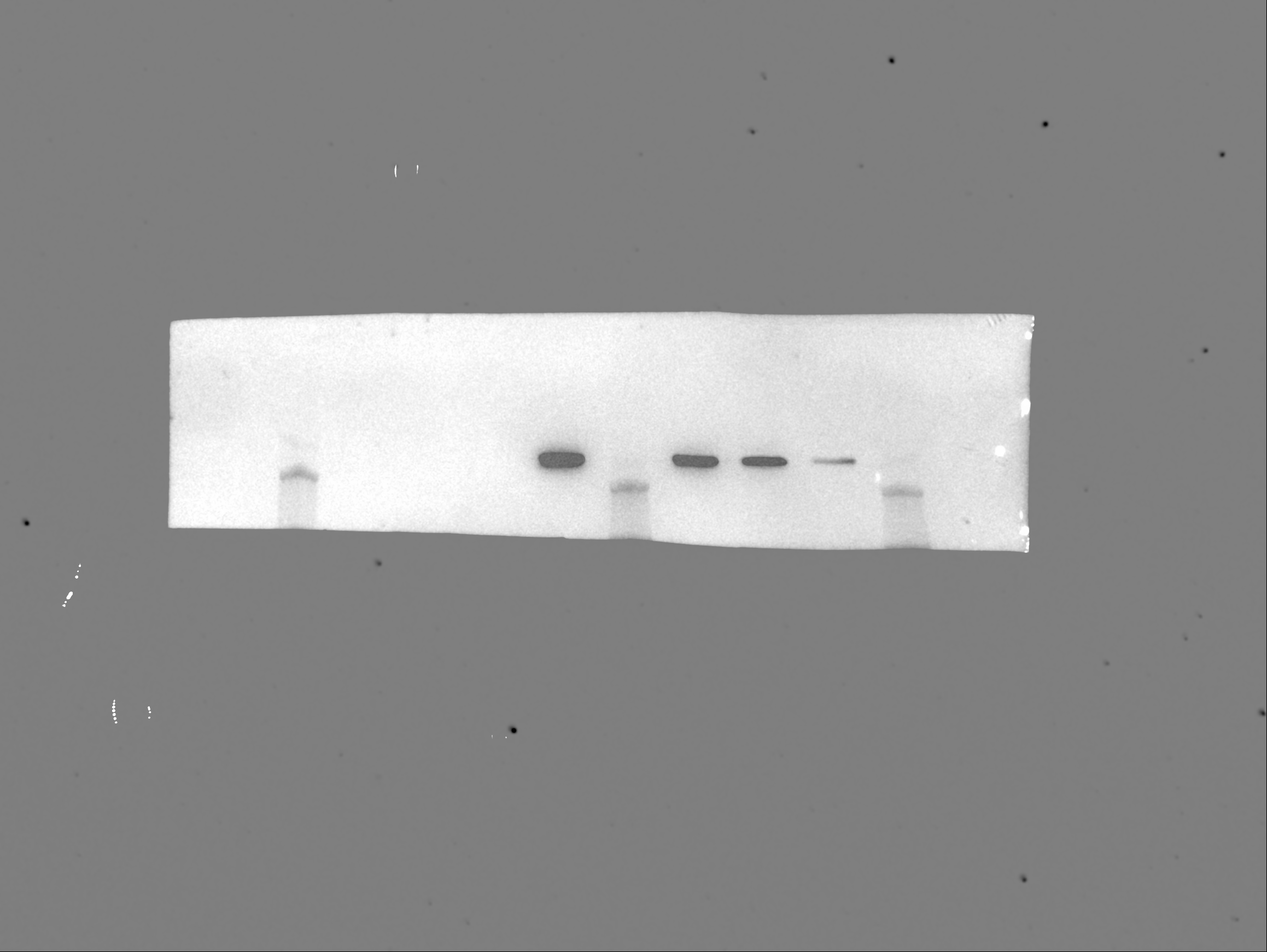

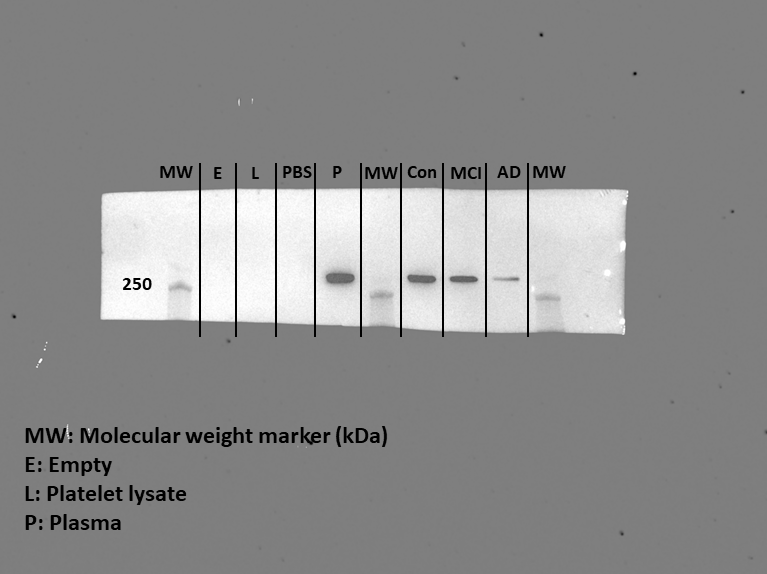


Western blot image – ALIX


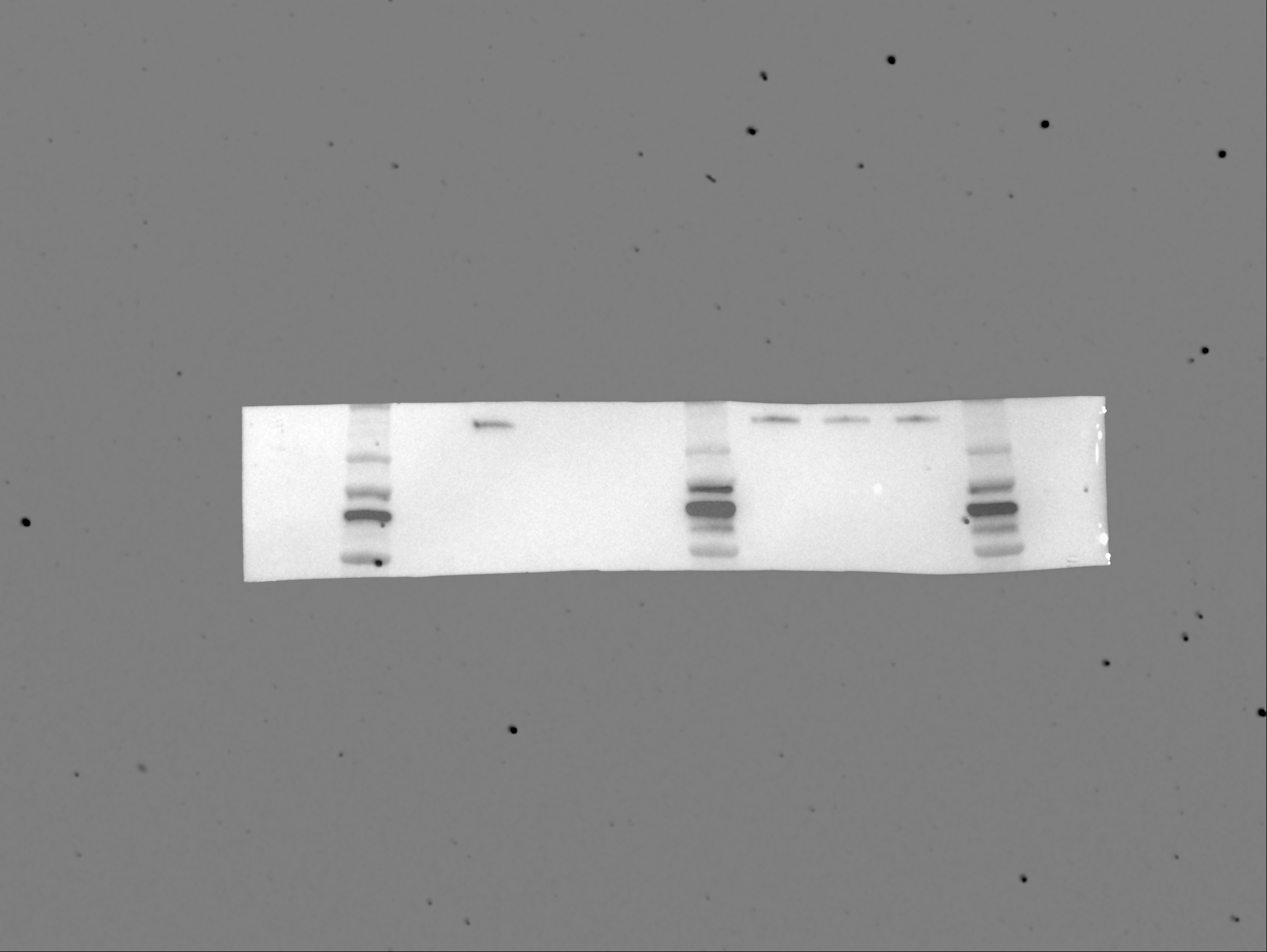

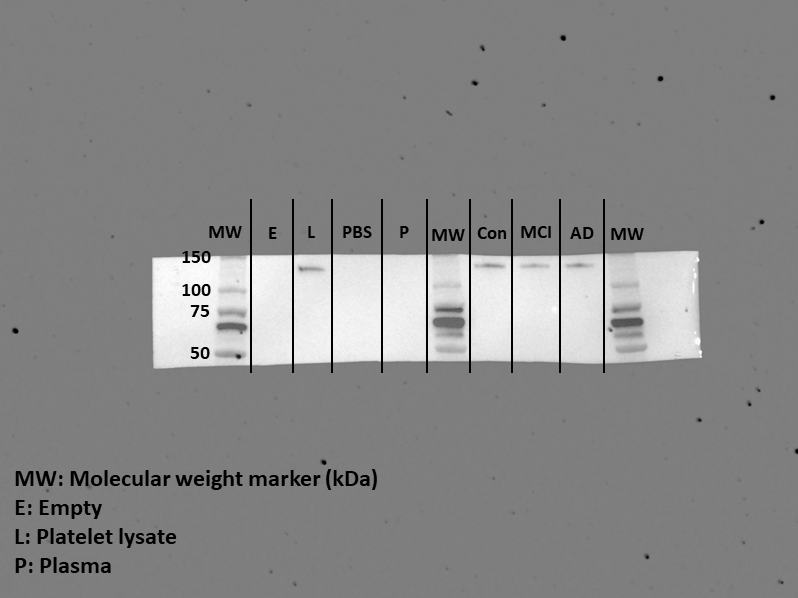


Western blot image – CD9


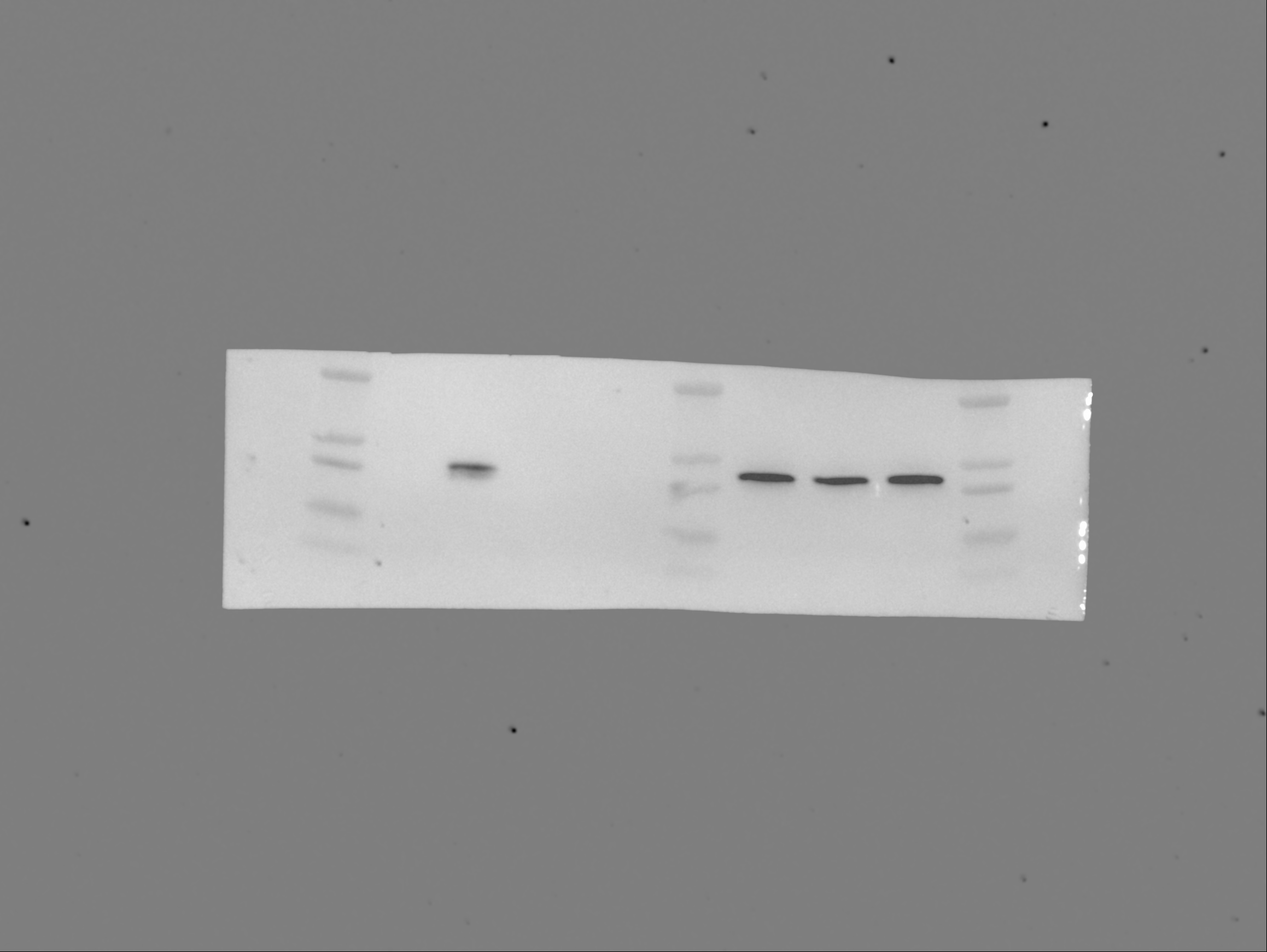

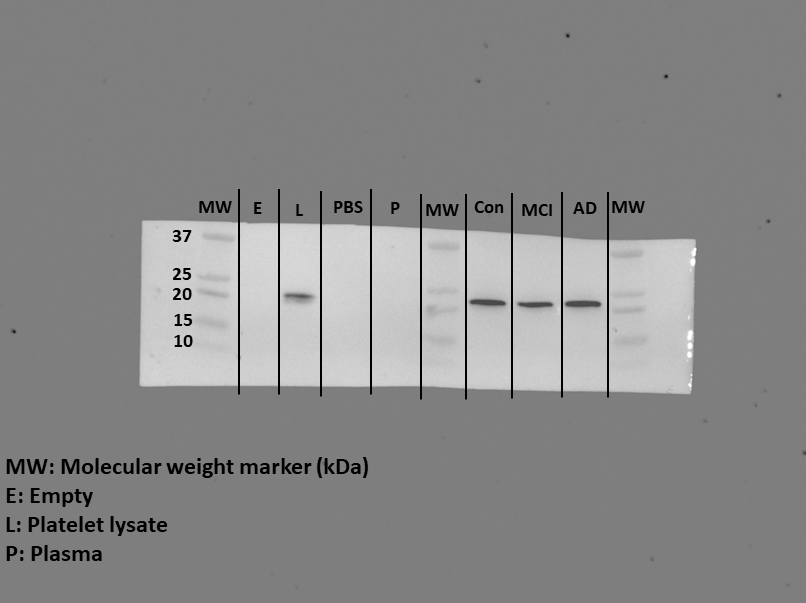


Transmission electron microscopy image – Healthy control sample


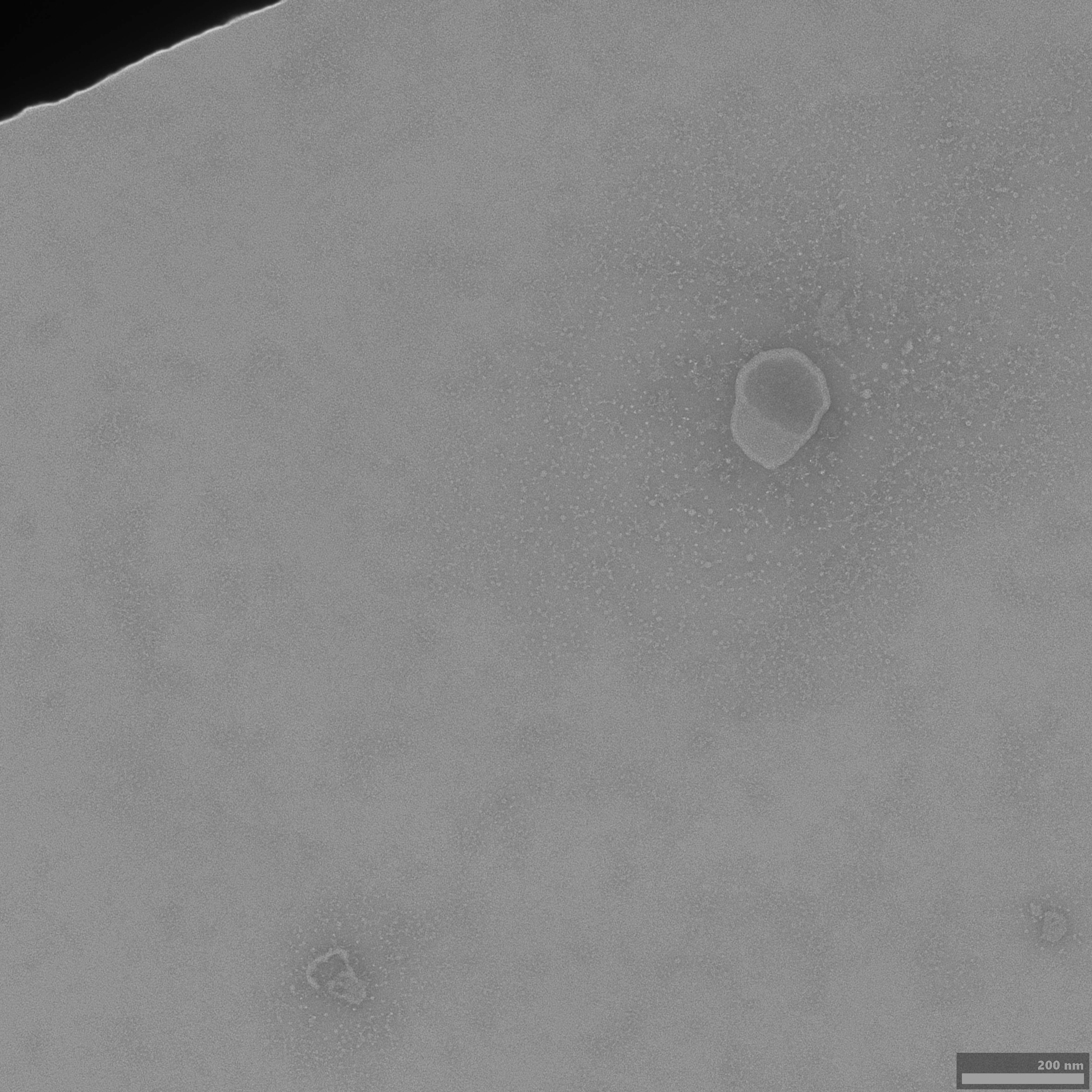


Immuno-electron microscopy image – Healthy control sample


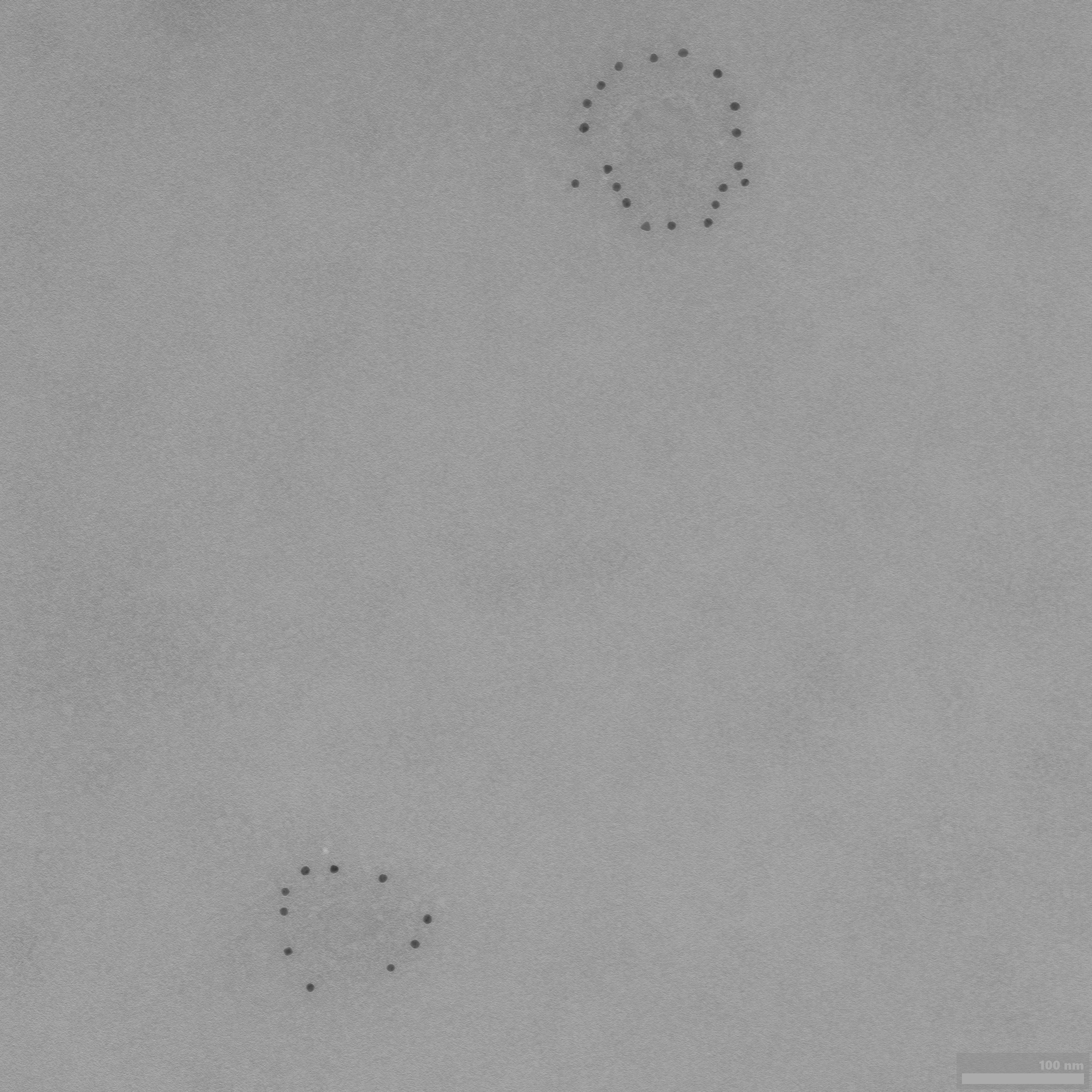


Transmission electron microscopy image – MCI patient sample


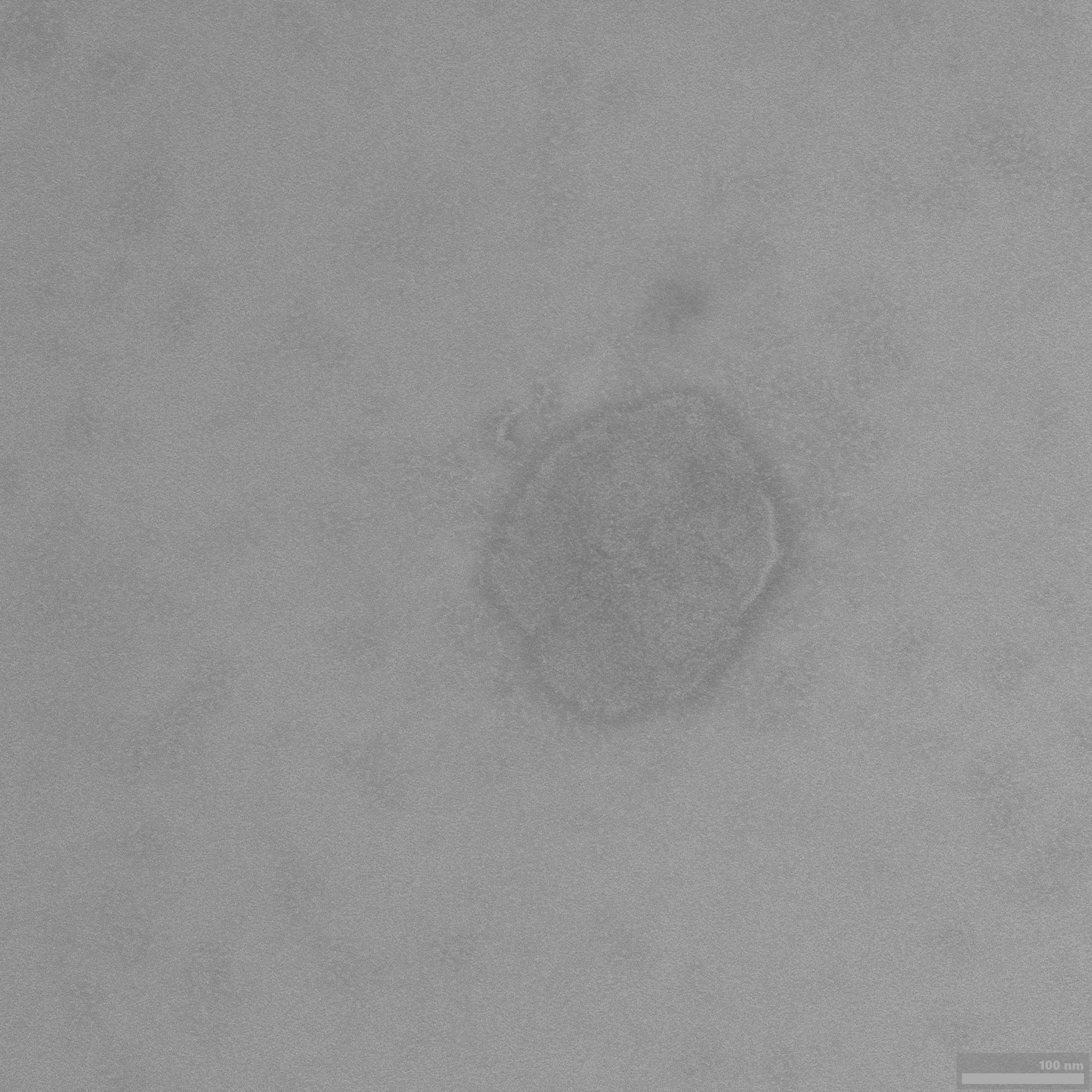


Immuno-electron microscopy image – MCI patient sample


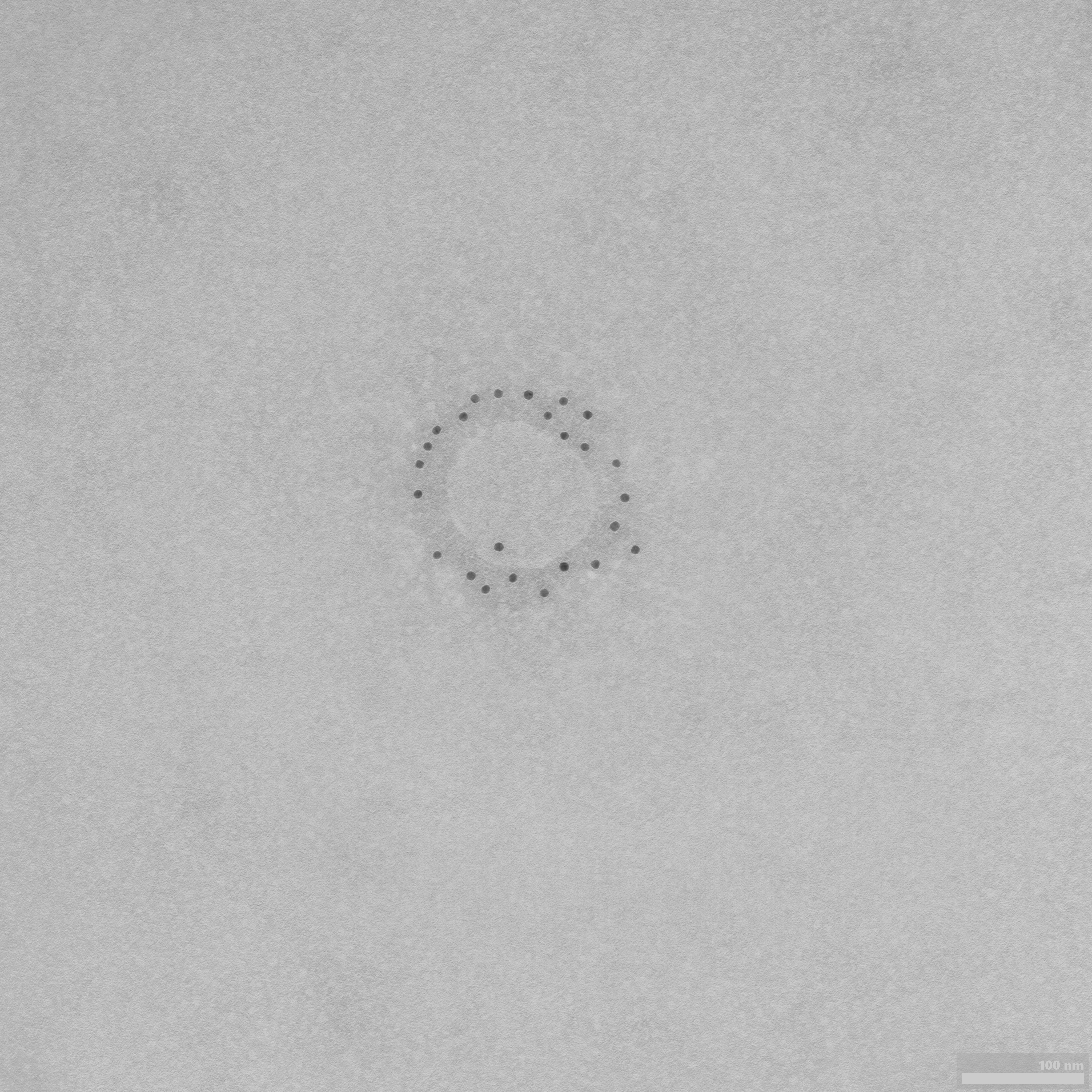


Transmission electron microscopy image – AD patient sample


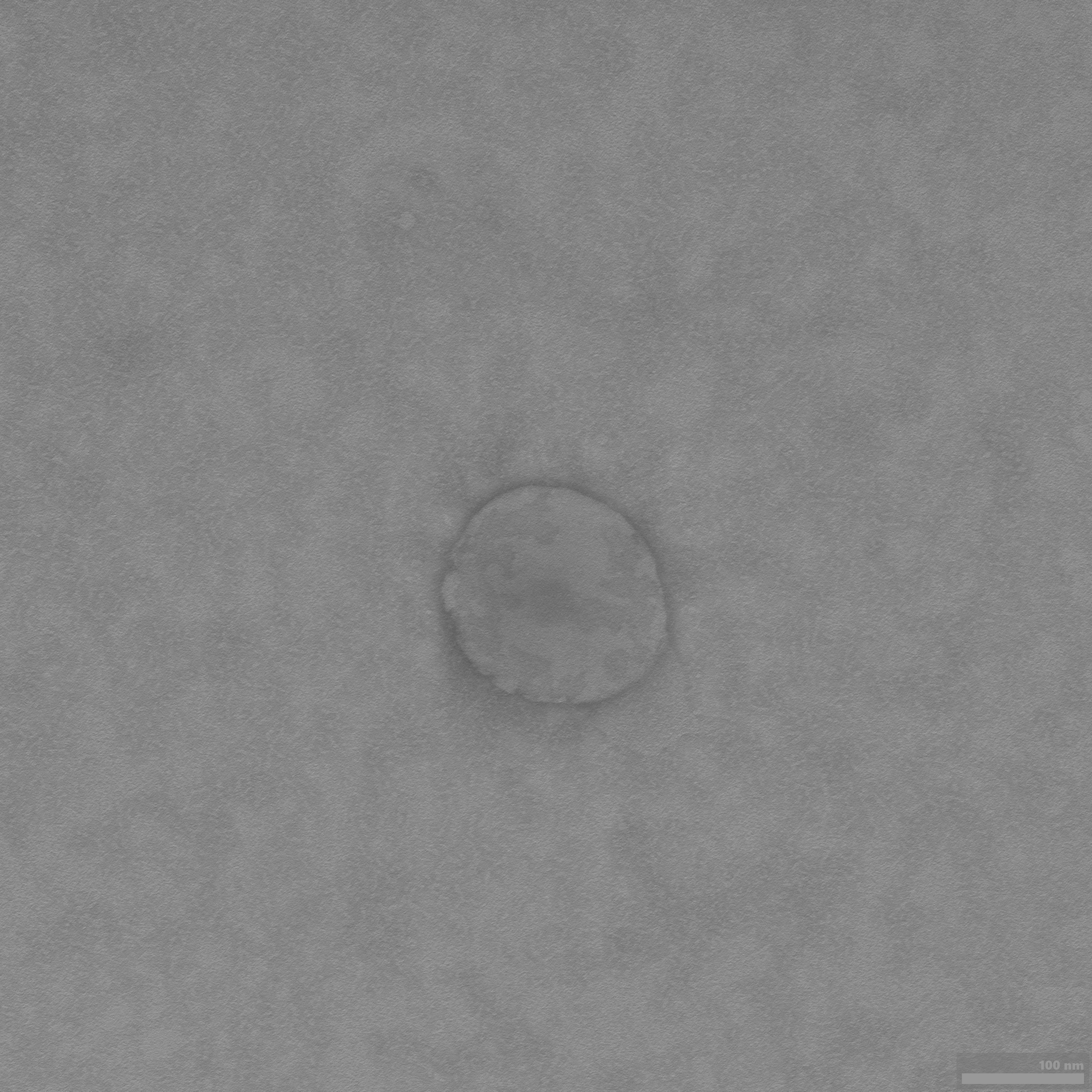


Immuno-electron microscopy image – AD patient sample


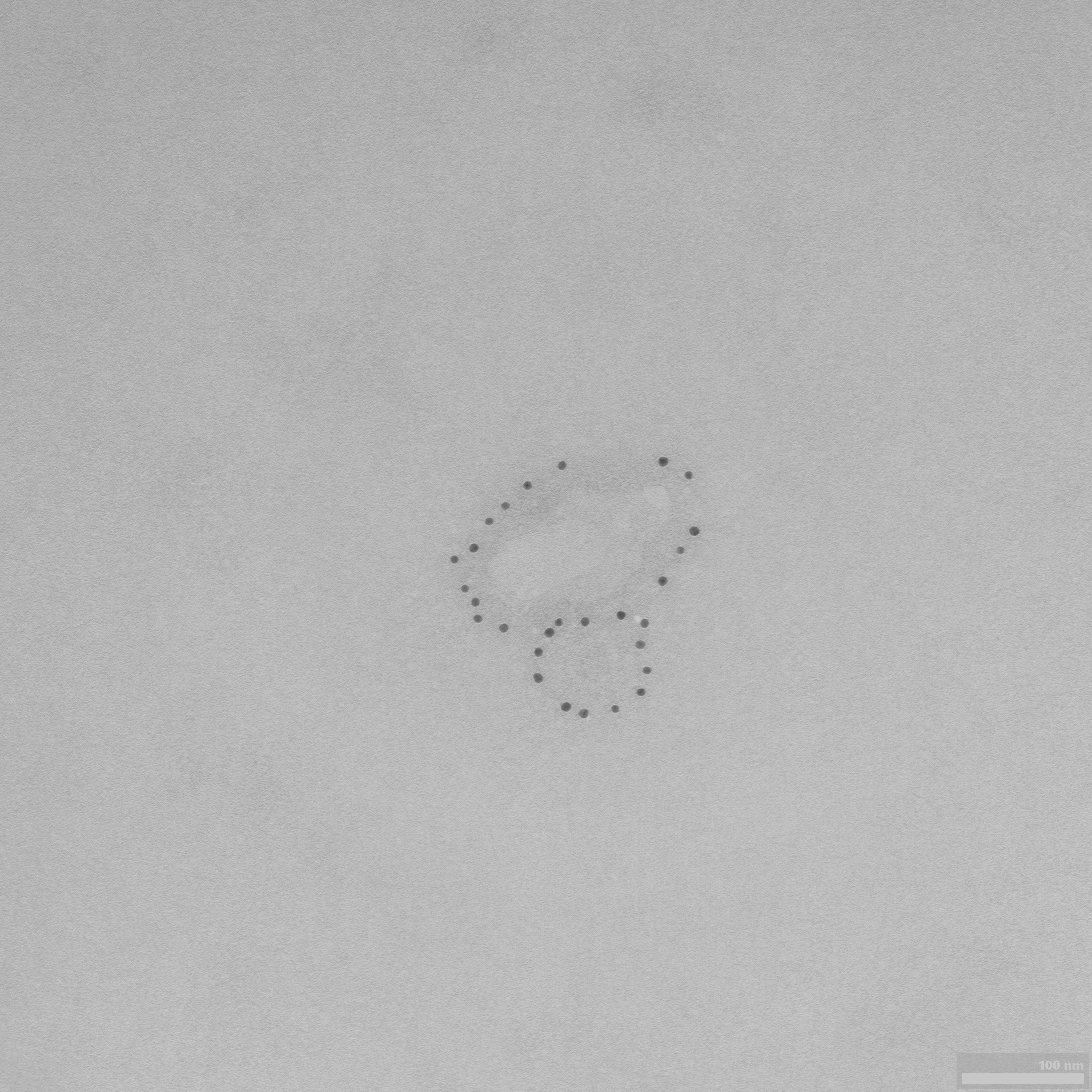


**Figure S2.** Principal component analysis with 100 % valid values.


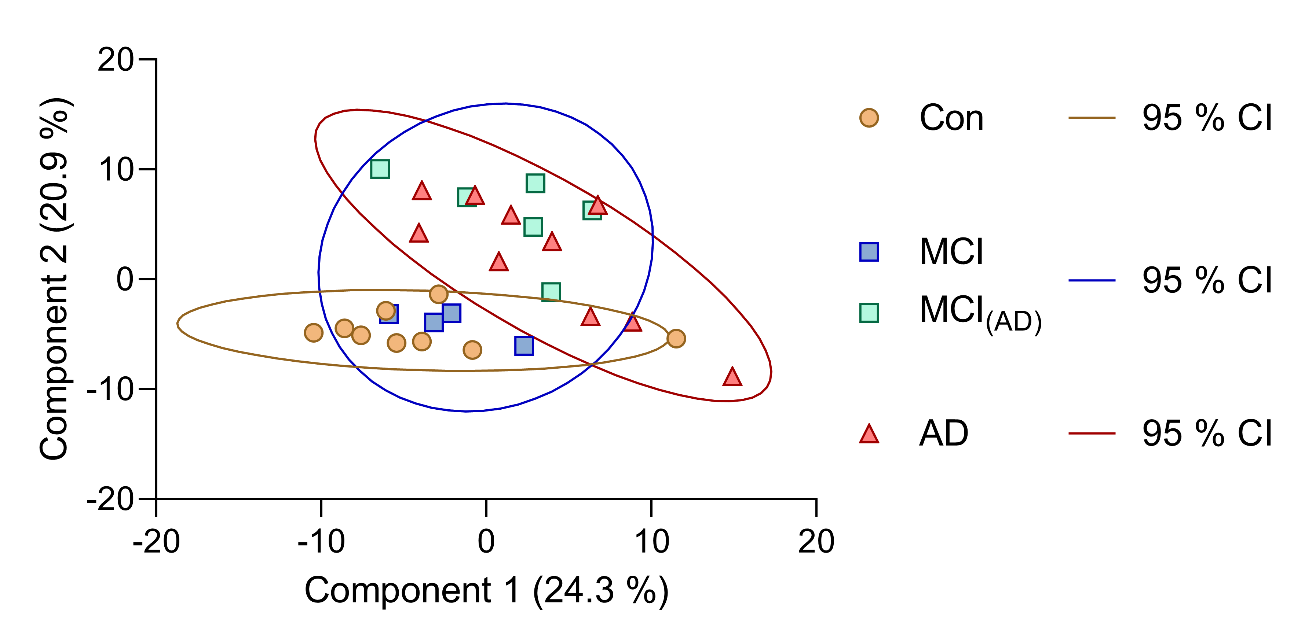


**Figure S3.** Plasma measurements of FXIII and ORM.


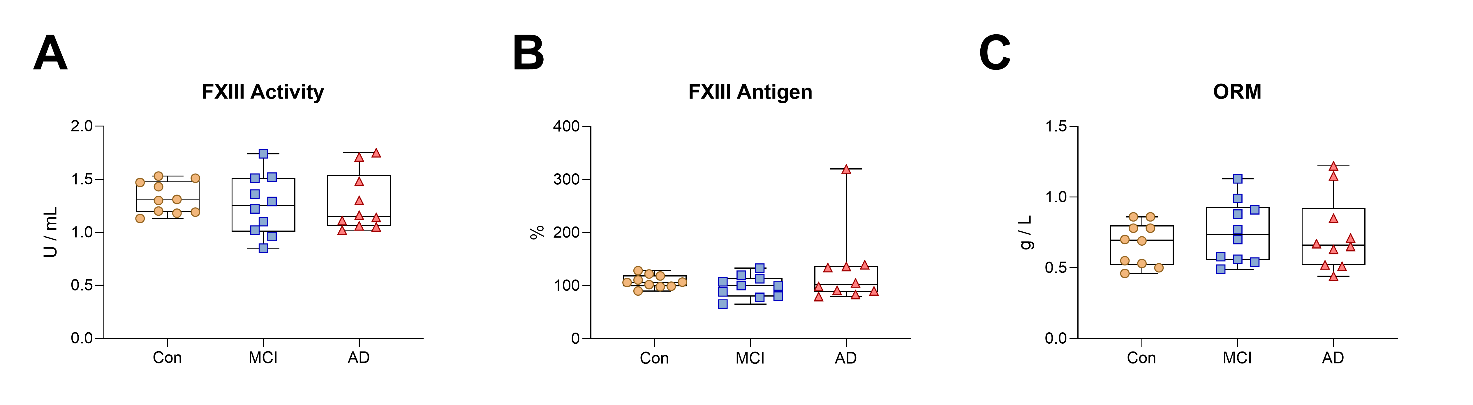

Supplement: Supplementary file 2 — Supplementary Information 2. [file 41598_2021_97969_MOESM2_ESM.docx]
